# QTL Mapping of Fiber Quality and Yield-Related Traits in an Intra-Specific Upland Cotton Using Genotype by Sequencing (GBS)

**DOI:** 10.3390/ijms19020441

**Published:** 2018-02-01

**Authors:** Latyr Diouf, Richard Odongo Magwanga, Wenfang Gong, Shoupu He, Zhaoe Pan, Yin Hua Jia, Joy Nyangasi Kirungu, Xiongming Du

**Affiliations:** 1 State Key Laboratory of Cotton Biology, Institute of Cotton Research, Chinese Academy of Agricultural Sciences, Anyang 455000, China; latyr@cricaas.com.cn (L.D.); richard@cricaas.com.cn (R.O.M.); gongwenfang@caas.cn (W.G.); heshoupu@caas.cn (S.H.); panzhaoe@caas.cn (Z.P.); jiayinhua@caas.cn (Y.H.J.); nyangasijoy@yahoo.com (J.N.K.); 2 Senegalese River Valley Development Agency (SAED), Saint-Louis Bp74, Senegal; 3 School of Physical and Biological Sciences (SPBS), Jaramogi Oginga Odinga University of Science and Technology (JOOUST), Main Campus, P.O. Box 210-40601, Bondo, Kenya

**Keywords:** QTL mapping, fiber quality, upland cotton, intra-specific, yield related traits, gene ontology, genotype by sequencing

## Abstract

Fiber quality and yield improvement are crucial for cotton domestication and breeding. With the transformation in spinning techniques and multiplicity needs, the development of cotton fiber quality and yield is of great importance. A genetic map of 5178 Single Nucleotide Polymorphism (SNP) markers were generated using 277 F_2:3_ population, from an intra-specific cross between two upland cotton accessions, CCRI35 a high fiber quality as female and Nan Dan Ba Di Da Hua (NH), with good yield properties as male parent. The map spanned 4768.098 cM with an average distance of 0.92 cM. A total of 110 Quantitative Traits Loci (QTLs) were identified for 11 traits, but only 30 QTLs were consistent in at least two environments. The highest percentage of phenotypic variance explained by a single QTL was 15.45%. Two major cluster regions were found, cluster 1 (chromosome17-D03) and cluster 2 (chromosome26-D12). Five candidate genes were identified in the two QTL cluster regions. Based on GO functional annotation, all the genes were highly correlated with fiber development, with functions such as protein kinase and phosphorylation. The five genes were associated with various fiber traits as follows: *Gh_D03G0889* linked to qFM-D03_cb, *Gh_D12G0093*, *Gh_D12G0410*, *Gh_D12G0435* associated with qFS-D12_cb and *Gh_D12G0969* linked to qFY-D12_cb. Further structural annotation and fine mapping is needed to determine the specific role played by the five identified genes in fiber quality and yield related pathway.

## 1. Introduction

Cotton is one of the most important natural fibers and oil crops in the world. Its annual global market value was estimated to be $630.6 billion in 2011 [1]. Cotton fiber is the primary raw material in the textile industry [2]. The advancements in techniques and diversified methods of spinning have made cotton fiber quality and related yield traits of paramount significance in breeding and production of cotton [3]. Fiber quality is determined by a number of factors such as fiber strength, fiber length, fiber micronaire and fiber color, while yield is mainly determined by lint quantity [4]. However, lint yield and fiber quality have been found to be negatively correlated [5,6], which has long been a critical issue in cotton breeding [7]. Recently, Shang et al. [8] identified 20 QTLs for fiber quality-related traits, however, four QTLs were validated. Moreover, five fiber quality traits were linked to 59 QTLs in an earlier report across five environments [9]. So far, few numbers of QTLs have been employed in marker-assisted selection (MAS) which is one of the enhanced breeding methods [10]. In all the identified and documented QTLs related to fiber and yield traits, most of them have been localized in a wide range of genomic regions and are often not stable across a wide genetic backgrounds [11]. Therefore, a dense interspecific map was generated, which included 2316 loci on the 26 cotton chromosomes in order to reduce and enhance accuracy in mapping [12]. However, these maps developed from interspecific hybridization have limited use in breeding due to limitation in controlling defective genes [2,5].

To overcome the inefficiency of maps developed from interspecific hybridization, it is therefore imperative to generate molecular maps based on an intraspecific population due to their ability to reduce the wide genome gap [2]. The employment of molecular marker techniques in cotton breeding through MAS and more advanced approaches such as genomic selection (GS) [13] would help break the bottleneck and, in turn, development of genetically advantaged genotypes. A small part of a DNA can be archived by reducing the complexity of the genome by restriction enzymes, such as genotyping-by-sequencing (GBS), the reduced-representation libraries (RRLs), restriction-site-associated DNA sequencing (RAD-seq) and next generation sequencing (NGS) [14].

The next-generation sequencing (NGS) of crop plant genomes have transformed the field of plant breeding. In the recent past, a lot of data generated has facilitated the discovery and use of large scale of single nucleotide polymorphisms (SNPs) in different genomes [15,16]. One of which was, genotype by sequencing (GBS), which holds the potential to narrow down the genotyping gap between references of large interest and mapping or breeding populations of local or specific interest [17]. GBS protocol techniques with their sample multiplicity have kept molecular research costs low while their output has diverse applications in many research areas, ranging from gene discovery to genomic-assisted breeding [18]. The ability of generating large amounts of unbiased markers in an inexpensive methods, has enabled GBS to become a more attractive approach to genotype and to construct high-resolution genetic maps, genomic selection and facilitated QTL mapping [19].

Mapping of QTLs has become an important technique to facilitate quantitative trait research and has been largely used in agricultural crops to map a number of beneficial agronomic traits including fiber quality and related yield traits.

In this investigation, a genetic map of 5178 SNP markers was generated using a 277 F_2:3_ intraspecific population developed from two tetraploid upland cotton accessions, mainly cultivated in China. CCRI35 with good fiber quality as female parent and Nan Dan Ba Di Da Hua (NH) known for high yield fiber as male parent. The map generated was employed to analyze QTLs related to fiber quality and yield related traits using QTL cartographer [20]. The aim of this study was to identify QTLs related to fiber quality, yield component traits, localize their position within the cotton genome and to identify the genes tightly linked to those QTLs. Findings of this research could provide valuable insights for breeders to develop cultivars with both traits, yield and quality fiber and enhance selection in cotton breeding.

## 2. Results

### 2.1. Phenotypic Variation between the Two Parents

**Figure 1:**
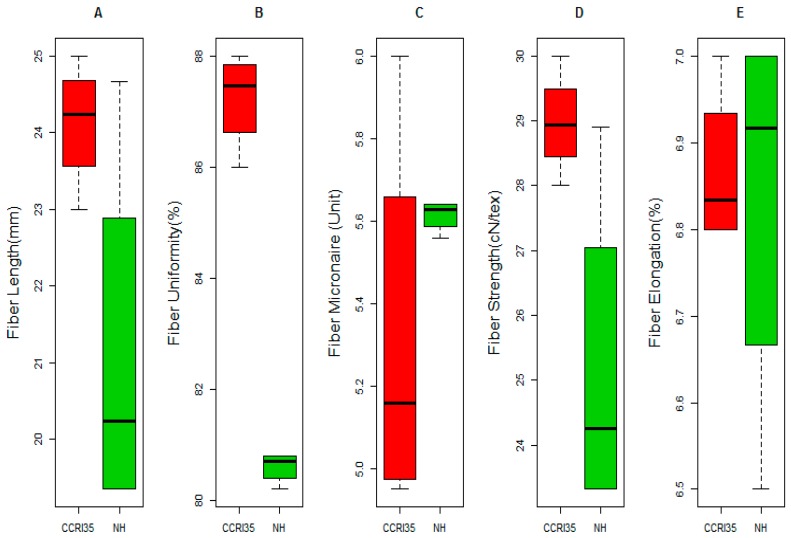
Phenotypic analysis of the two parents for fiber quality and yield-related traits; (**A**) Fiber Length (mm); (**B**) Fiber Uniformity (%); (**C**) Fiber Micronaire (Unit), (**D**) Fiber Strength (cN/tex); (**E**) Fiber Elongation (%).CCRI35: female parent, NH: male parent.

**Figure 2:**
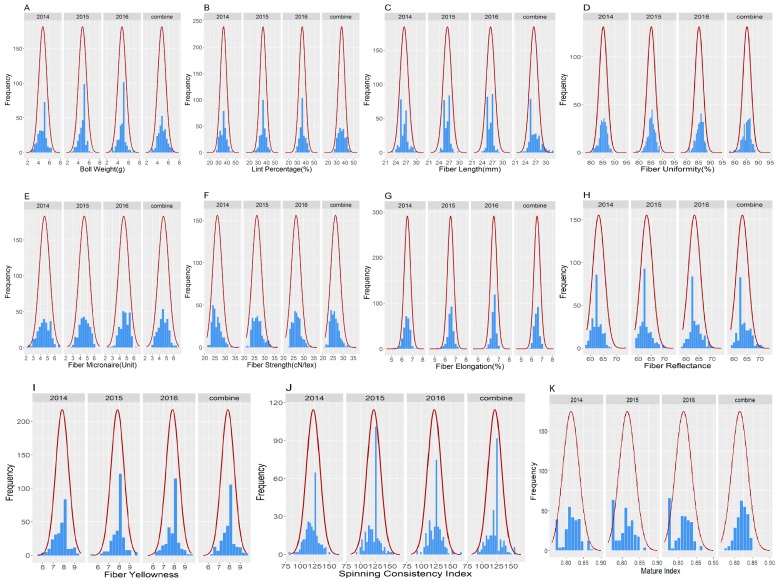
Frequency distribution of the 11 traits for fiber quality traits in F_2:3_, (**A**) Boll Weight (g); (**B**) Lint Percentage (%); (**C**) Fiber Length (mm); (**D**) Fiber Uniformity (%); (**E**) Fiber Micronaire (Unit); (**F**) Fiber Strength (cN/tex); (**G**) Fiber Elongation (%); (**H**) Fiber Reflectance; (**I**) Fiber Yellowness; (**J**) Spinning Consistency Index; (**K**) Mature Index.

In the determination of phenotypic variation of the 11 measured traits, Boll weight (BW), lint percentage (LP), fiber reflectance (FR), fiber yellowness (FY), spinning consistency index (SCI), and mature index (MI) were not used in analysis of the phenotypic variation between the parental lines due to the huge missing data throughout the phenotyping periods. The five traits used were fiber length (FL), fiber uniformity (FU), fiber strength (FS), fiber micronaire (FM) and fiber elongation (FE). FL, FU and FS showed significant differences between the parental lines. All traits were higher in CCRI35 than NH with exemption of FE which was higher in NH. In addition, no significant difference was noted between the two parental lines for fiber micronaire (FM) and fiber elongation (FE), Figure 1. However, there was a wide range of phenotypic variation among the F_2:3_ population, with respect to all the measured traits; BW, LP, FL, FU, FM, FS, FE, FR, FY, SCI and MI. Across the three environments, 2014, 2015 and 2016 all the traits showed normal segregation with normalized distribution patterns (Figure 2).

### 2.2. Correlation Analysis

**Figure 3:**
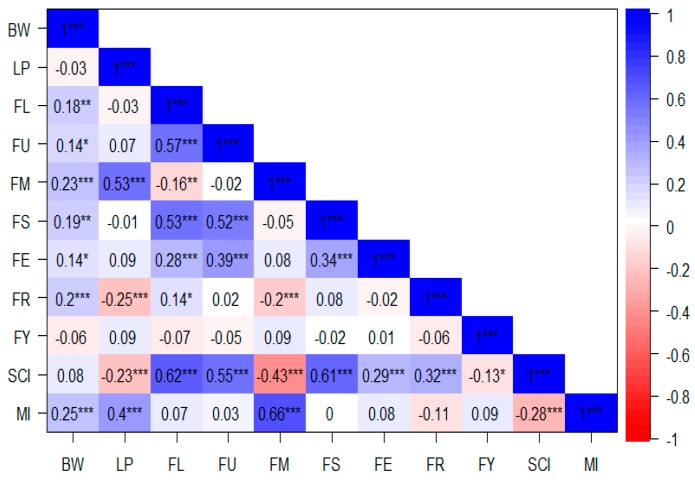
Pearson’s correlation of the 11 traits for the F_2:3_ in three environments. *, **, ***: significant levels of 0.5, 0.01 and 0.001 respectively. BW: Boll weight; LP: lint percentage; FL: fiber length; FU: fiber uniformity; FM: fiber micronaire; FS: fiber strength; FE: fiber elongation; FR: fiber reflectance; FY: fiber yellowness; SCI: spinning consistency index; MI: mature index. For the units, see in Figure 1 and Figure 2.

To determine the correlations among different traits, a Pearson’s correlation coefficient on yield-related and fiber quality trait was done using “Performance Analytics” package with Chart correlation function in R software version 3.4.2 [21]. Significant and positive correlations were noted between: BW with FL, FU, FM, FS, FE, FR, and MI; LP with FM and MI;FL with FU, FS, FE, FR, and SCI; FU with FS, FE, and SCI; FM with MI; FS with FE and SCI; FE with SCI and finally FR with SCI. Negative correlations were observed between: LP with FR and SCI; FL with FM; FM with FR and SCI; FY with SCI and finally SCI with MI (Figure 3). However, no significant correlation was noted between the other traits.

### 2.3. ANOVA, Broad Sense Heritability and Phenotypic Analysis of Fiber Quality for the Two Parents and the F_2:3_ Population

The ANOVA result revealed significant differences between the genotypes, environment and their interactions for all the traits (Table 1).

The broad sense heritability was much higher for the fiber quality traits as opposed to yield-related traits. The highest broad sense heritability was observed with fiber micronaire (FM), with 92.4% while the lowest broad sense heritability was observed in fiber elongation (FE) with 61.8%.

### 2.4. GBS Genotyping, SNP Detection and Annotation

The genotypic data for the entire population was developed by use of the genotyping by sequencing (GBS) technique. Fifteen (15) individuals of each of the parents were sequenced and mapped on to the reference genome, which we obtained from the cotton research institute (available online: http://mascotton.njau.edu.cn). We obtained a total of 20,542,731 and 20,244,825 reads for CCRI35 and NH, respectively. An average of 80,372 and 112,128 SNPs were eventually identified for the female parent (CCRI35) and the male parent (NH), respectively, with an enzyme digestion efficiency of 99%. In genotyping the F_2:3_ population, the enzyme efficiency was slightly lower compared to its efficiency in the parents, with efficiency of 98.9%. The overall mapped reads for the population and the two parents were 1,507,193,217, with an average of 4,909,424.16 mapped reads per individual which correspond to nearly 180.889 Gb of clean bases. The clean reads obtained were equivalent to 80.42-fold haploid genome coverage of raw paired-end Illumina reads by sequencing whole genome shotgun (WGS) libraries of homozygous cv. “TM-1” compared to Li et al. [22] in their study which generated a total of 445.7 Gb of clean reads translating to about 181-fold haploid genome coverage of raw paired-end Illumina reads by sequencing whole genome shotgun (WGS) libraries of homozygous cv. “TM-1” with fragment lengths ranging from 250 bp to 40 kb. The average GC content of the sequences was 38.25%, with a Q20 score of 94.66%. The parental lines were genotypes such as AC and AA, in which the female parent CCRI35 was heterozygous while the male parent (NH) was homozygous. The total resulting SNPs markers were 103,381 markers which were used to carry out further analysis. We assessed the distribution of the alleles across the F_2:3_ population, and those markers which had a coverage threshold of 75% were filtered out, eventually, 34,090 markers were used. Markers with significant distortion (*p* < 0.001) were filtered and 6405 markers were retained with the purpose of determining bin markers.

### 2.5. Construction of the Linkage Maps

**Figure 4:**
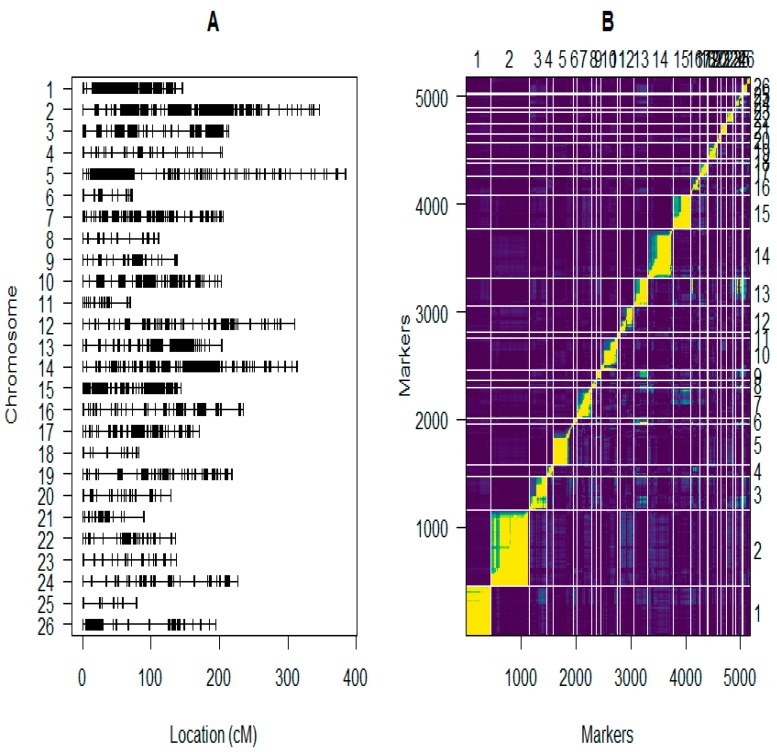
(**A**) Genetic linkage map constructed using the F_2:3_ Population; (**B**) Plot of estimated recombination fractions of all markers used in the F_2:3_ population. *X* and *Y* axis are the markers and *Z* is the linkage groups (LGs).

In the construction of the linkage groups, we used 6405 markers (Table S1) and phenotypic data of the F_2:3_ population developed from an intra-specific cross of two tetraploid upland cottons were utilized for developing the intra-specific linkage map. A total of 5178 GBS markers were used for mapping the F_2:3_ population, all the distorted markers were filtered out, the linkage groups were generated by the use of Join Map 4.0 [23]. Twenty six (26) LGs were generated from 5178 markers (Figure 4A and Figure S1, Table 2 and Table S2). Markers in linkage groups were ordered, rippled, and re-ordered according to pairwise recombination fractions, LOD scores (Logarithm of Odds) and linkage group length (Figure 4B). The 26 LGs were designated as A01 to A13 for A_t_ sub-genome and D01 to D13 for D_t_ sub-genome. The map generated had a map distance of 4768.098 cM, higher than the most current upland cotton linkage map with a map distance of 4450 cM [24]. The average distance between adjacent markers was 0.92 cM, the marker distances were narrowed in the map generated compared to earlier maps with 1.7 cM between adjacent markers [24]. The At sub-genome spanned 2611.43 cM, with a total of 3313 markers in the 13 linkage groups, with an average distance of 0.79 cM, while in D_t_ sub-genome, thirteen linkage groups comprised of 1865 markers spanning a distance of 2156.67 cM, with an average of 1.156 cM. The maximum gap between adjacent loci was 26.598 cM and 30.082 cM in A_t_ and D_t_ respectively, affirming the genome lengths between A_t_ and D_t_ [24] (Table 2). Chromosomes; A02, D02, A01, A05, A03, D01 and A10 exhibited higher marker loci with higher recombination frequency compared to the rest of the chromosomes such as D06 and D13 (Figure 4A,B). The chromosome with the highest marker loci was chromosome A02, 705 loci with map distance of 346.314 cM and an average distance of 0.49 cM, while the lowest marker loci was detected in chromosome D06 with only 16 markers, and a total length of 79.084 cM (Figure 4B).

### 2.6. Identification of Consistent and Clustering QTLs for Yield Related and Fiber Quality Traits

Thirty (30) QTLs were consistent among all the 110 QTLs identified for 11 traits in at least two environments (Table 3 and Figure S1). The 30 consistent QTLs were located on 16 chromosomes; A02 (2), A03 (1), A05 (2), A09 (3), A10 (2), A12 (1), D01 (1), D02 (1), D03 (4), D04 (1), D05 (2), D08 (2), D10 (2), D11 (1), D12 (4), and D13 (1). The distribution of the QTLs within the identified chromosomes, exhibited multiple position as illustrated in Figure S1 and Table S2 and Table 3. Of the 30 detected QTLs, 11 were localized on A_t_ sub-genome while the remaining 19 were mapped on the D_t_ sub-genome. The contributions of the parents toward the QTLs: 19 QTLs were linked to the good fiber quality parent (CCRI35) while only 11 QTLs were contributed by the high yield fiber parent (NH). Only16 chromosomes out of 26 were found to harbor consistent QTLs for ten traits except MI (Mature Index) for yield-related and fiber quality (Table S2 and Table 3).

Four types of gene actions were revealed by the genetic effects of which one gene exhibited dominant effects (De), four partial dominances (PD), 20 over dominances (OD) and five additive effects (Ae). OD was observed for most of the traits in response to yield-related and fiber quality traits.

**Figure 5:**
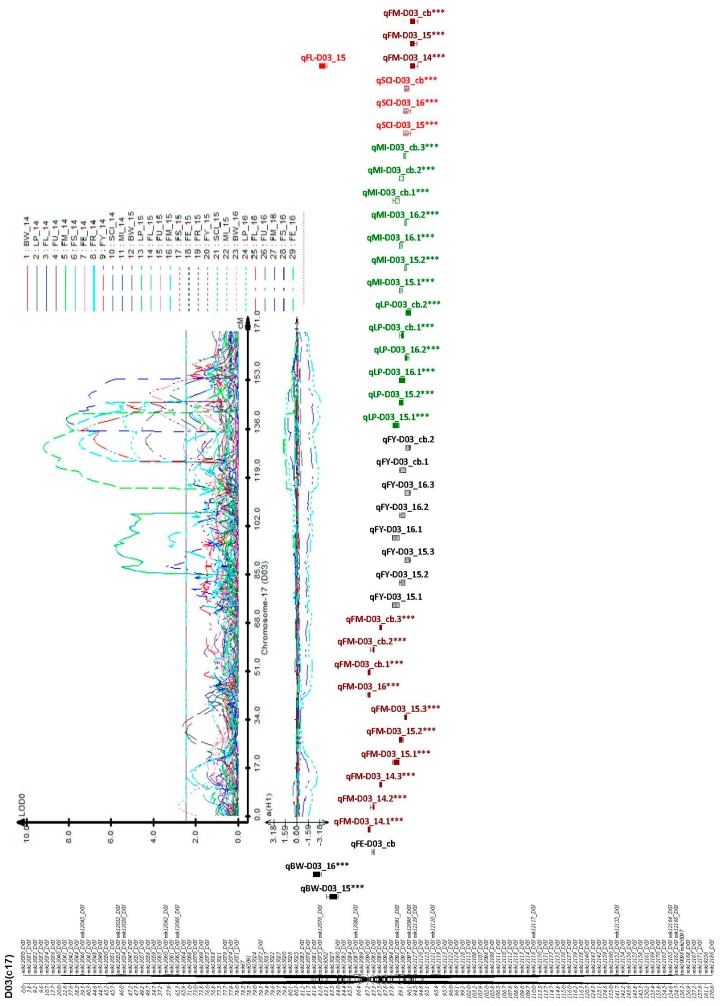
Clustered QTLs identified in D03 (c17) of yield-related and fiber quality traits. Bars and lines on the right-hand side of the linkage groups show the QTL likelihood intervals. Map distances in centiMorgan (cM) are indicated on the left-hand side of the linkage groups. For trait meanings, see Figure 1 or Figure 2, *** asterisk means the QTL is consistent.

**Figure 6:**
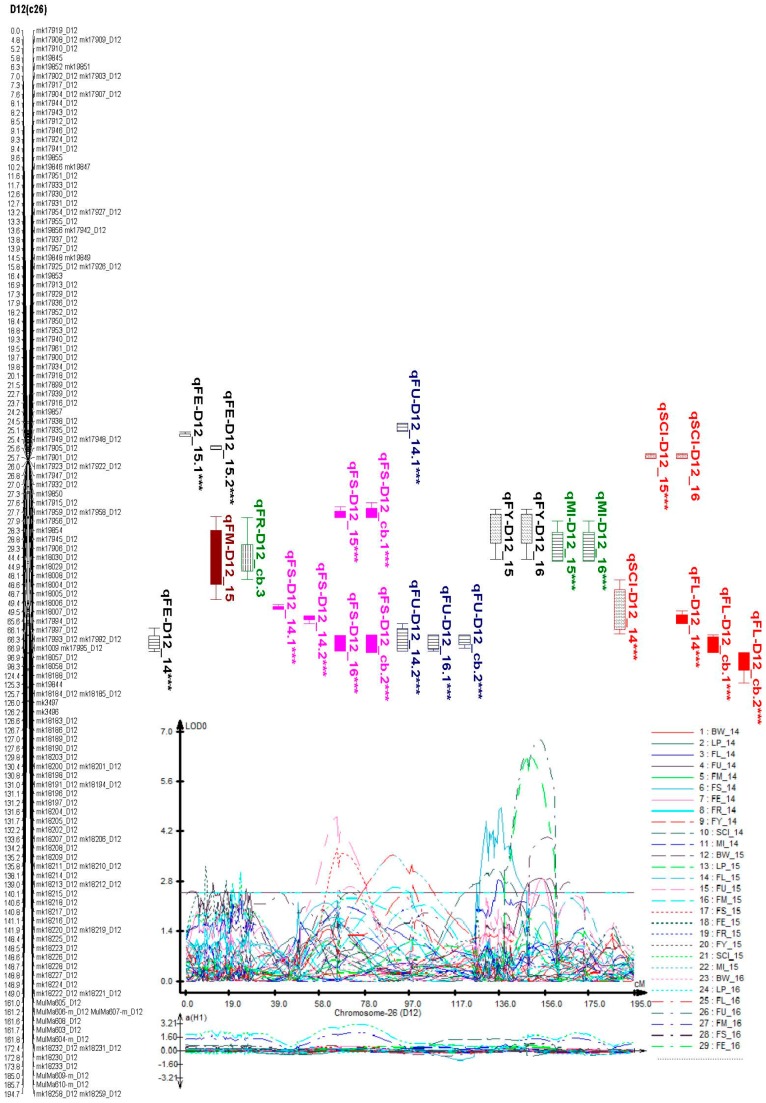
Clustered QTLs identified in D12 (c26) of yield-related and fiber quality traits. Bars and lines on the right-hand side of the linkage groups show the QTL likelihood intervals. Map distances in centiMorgan (cM) are indicated on the left-hand side of the linkage groups. For trait meanings, see Figure 1 or Figure 2, *** asterisk means the QTL is consistent.

The highest percentage of phenotypic variance explained by a single QTL was 15.45%. The highest percentage of phenotypic variance was noted in lint percentage (LP), with a range of 10.03–15.46%. The distribution of the QTLs within the identified chromosomes, exhibited multiple positions in some chromosomes; A02, A03, A09, A10, D01, D03, D05, D08, D12, and D13 as illustrated in Table S2 and Table 3 and Figure S1. Moreover, a total of two important clusters with more than three traits per region, with high broad sense heritability and high percentage of phenotypic variation were identified as D03 (c17) and D12 (c26), which we designated as cluster 1 and cluster 2, respectively (Table 3, Figure 5 and Figure 6).

### 2.7. The Gene Ontology Enrichment Analysis Based on QTL Clusters

Based on phenotype variation and QTL frequency, D_t_-sub genome of the whole tetraploid chromosomes, harbored the highest number of stable QTLs with the highest level of phenotypic variation. In lieu of this, chromosome 17 (D03) and chromosome 26 (D12) had two clusters with four QTLS in each. Within the two cluster regions, we were able to mine the putative genes which could be having a role in fiber and yield-related traits. In cluster 1 (Chr17, D03), 136 genes were obtained, in which 14 were found to be highly expressed based on the RNA sequence while in cluster 2 (Chr26, D12), a total of 1280 genes were mined, out of which 153 were highly expressed at various stages of fiber development, 5 DPA, 10 DPA, 20 DPA and 25 DPA.

**Figure 7:**
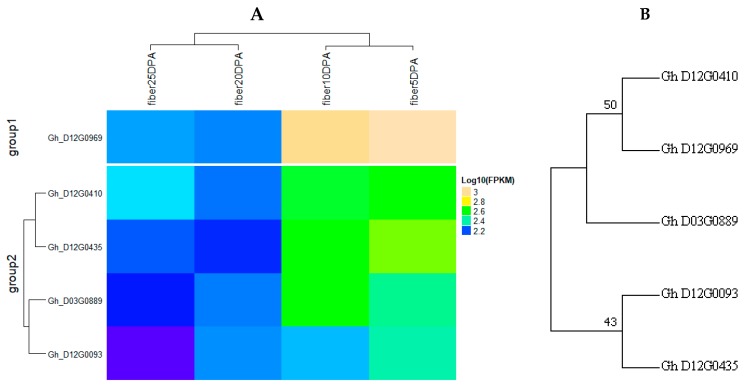
(**A**) Candidate genes involved in yield and/or fiber quality traits in this study; (**B**) phylogenetic tree analysis of the five involved genes in yield related and fiber quality traits of upland cotton.

**Figure 8:**
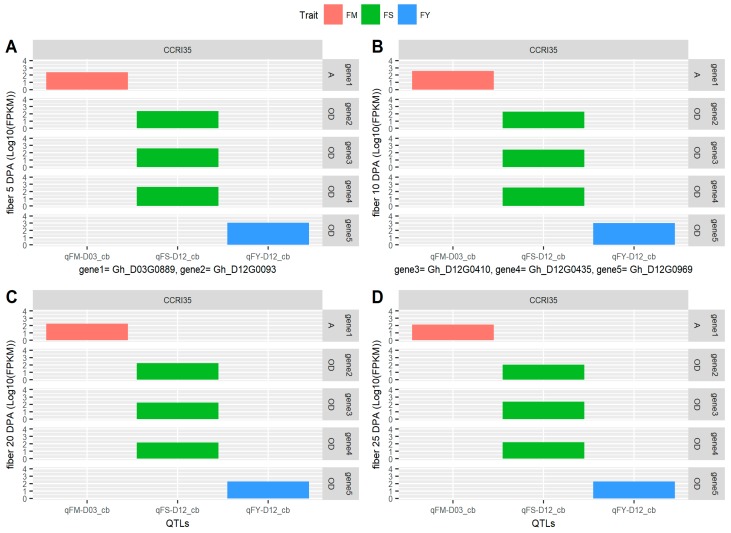
The five genes with highest expression in different stages of fiber development. *x* axis: QTLs for fiber Quality; CCRI35: good fiber quality parental line; *y* axis: fiber DPA (Log10(FPKM)); FM: fiber micronaire (%); FS: fiber strength (cN/tex); FY: fiber yellowness; *Gh_D03* or *Gh_D12* are genes identified with high expression and involved in fiber development; cb: combine analysis; 0 <Ae (additive effect) <0.20; OD (over dominance) > 1.20; (**A**–**D**) are respectively fiber expression at 5, 10, 20 and 25 DPA. For trait meanings, see Figure 1 or Figure 2.

Moreover, in order to identify the set of the most robust candidate genes for yield-related traits and fiber quality; we mainly focused on the 153 highly expressed genes as obtained from “TM-1”_RNA-seq data (available online: http://mascotton.njau.edu.cn). Out of 153 highly expressed genes, five showed high level of expression across the various stages of fiber development, and therefore, the five genes could be the potential candidate genes with greater roles in the regulation of various fiber traits Table S3. Furthermore, all the five genes were localized in different positions of the genome: one gene (*Gh_D03G0889*) was located in cluster 1 (D03 (Chr 17)) within the marker mk12119_D03 (30,535,745 bp) to marker mk12123_D03 (30,566,883 bp), the trait localized in this region was fiber micronaire (FM); while the other three genes: *Gh_D12G0093*, *Gh_D12G0410*, and *Gh_D12G0435* were localized in cluster 2 (D12 (c26)) within the marker mk19853 (101,319 bp) to marker mk17913_D12 (13,479,261 bp), the trait localized in the genome region was fiber strength (FS). Finally, the fifth gene, *Gh_D12G0969* was also mapped in cluster 2 (D12 (Chr 26)), from marker mk1009 (18,989 bp) to marker mk17992_D12 (37,732,030 bp), the trait localized in that area was fiber yellowness (FY). Based on the expression profile and GO functional annotation, these five genes were therefore found to be the most robust and possibly the putative candidate genes for fiber quality and yield related traits (Table S3, Figure 7 and Figure 8).

Based on GO enrichment analysis, the five highly up regulated genes were as follows: *Gh_D03G0889* was mainly involved in molecular function and biological processes, such as, up regulation of translational elongation (GO: 0003746), poly-A RNA binding (GO: 0003723), ribosome receptor activity (GO: 0043022), hypusine anabolism (GO: 0008612), translation elongation factor (GO: 0003746), regulation of translation elongation (GO: 0045901) and regulation of translation termination (GO: 0045905). The second gene, *Gh_D12G0093* was involved only in molecular function, protein amino acid binding (GO: 0005515). The third gene, *Gh_D12G0410* was involved in all the GO functional annotation, in biological process, it was mainly involved in translation elongation (GO: 0006414), molecular function, it was mainly involved in translation elongation factor activity (GO: 0003746) and protein binding (GO: 0005515) while in cellular component, it was found to be involved in eukaryotic translation elongation factor 1 complex (GO: 0005853). The fourth gene, *Gh_D12G0435,* had no functional annotation, however it was found to function in nucleoside diphosphate kinase activity and the last gene, *Gh_D12G0969*, functions both in biological process and molecular function, nucleoside diphosphate kinase activity (GO: 0004550), nucleoside diphosphate phosphorylation (GO: 0006165), GTP biosynthetic process (GO: 0006183), UTP biosynthetic process (GO: 0006228), CTP biosynthetic process (GO: 0006241) and ATP binding (GO: 0005524). In relation to gene action analysis, the five putative and robust genes with direct role in fiber development in cotton were all contributed by the female parent, CCRI35, known for its superior fiber quality (Table S3 and Figure 7 and Figure 8). The five genes had similar sequences based on phylogenetic tree analysis; the same was affirmed by their expression profile and all from D_t_-sub genome. High quality fiber attributes are highly linked to the D-genome of the diploid cotton such as *G. barbadense*, and being tetraploid cotton originated from the polyploidization of the A and D genomes of the diploid cotton.

## 3. Discussion

The determination of stable QTLs for superior agronomical traits and the construction of a high-resolution map are crucial for MAS. Several intra-specific genetic maps have been generated and used for QTL detection related to fiber and yield components [2]. Even though these maps have been used, they are limited in scope and accuracy due to huge marker intervals and narrow genome coverage. The greatest impediment in the construction of a high-resolution map in intraspecific crosses is due to low rate of polymorphism within *G. hirsutum* and the presence of fixed homozygous genetic blocks [11,25]. Therefore, there is a need to find additional markers to fill in the gaps in the genetic map [11]. In this current research, a genetic map consisting of 5178 SNP markers obtained through the GBS technique was developed using a 277 F_2:3_ population derived from an intra-specific cross. In addition, the contrasting difference between the two parental lines used in this investigation could be explained based on inherent genetic characteristics. The male parent is known for superior agronomic traits such as early flowering and the ability to generate a high percentage of fruits with large size, while the female parent is known for superior fiber traits. Fiber length (FL), fiber uniformity (FU), fiber micronaire (FM), and fiber strength (FS) showed significant differences between the two parental lines. These traits were attributed to CCRI35, except FE which was linked to NH. There was no significant difference noted between the two parents for FE and FM. This result confirmed the good quality fiber trait of the female parent, CCRI35, compared to the male, NH.

In addition, there was a wide range of phenotypic variation among the F_2:3_ population, with respects to the following measured traits: BW, LP, FL, FU, FM, FS, FE, FR, FY, SCI, and MI. In the three environments, all traits exhibited normal segregation patterns, with equal distribution. The low absolute values for skewness and kurtosis showed that these traits had normal distribution. In addition, in the F_2:3_ population, the maximum phenotypic data values in all the variables were much higher than in CCRI35, the parent known for superior fiber traits, fiber length (FL), fiber uniformity (FU), fiber micronaire (FM), fiber strength (FS), and fiber elongation (FE). This finding showed that all traits were transgressively segregated in the F_2:3_ population. Previous research reported that transgression was the difference observed between the mapping parents of upland cotton [11,26,27,28].

Furthermore, positive correlations were noted between the following traits: boll weight (BW) with fiber length (FL), fiber uniformity (FU), fiber micronaire (FM), fiber strength (FS), fiber elongation (FE), fiber reflectance (FR), and mature index (MI); lint percentage (LP) with FM, FL with FU, FS, FE, and spinning consistency index (SCI); FU with FS, FE, and SCI; FM with MI; FS with FE, and SCI; FE with SCI and finally FR with SCI. However negative correlations were observed in the following traits: LP with FR, and SCI; FL with FM; FM with FR, and SCI; finally, SCI with MI. This result is consistent with those from Jamshed et al. [11] which showed that positive correlations were observed between: fiber elongation (FE), fiber length (FL), fiber strength (FS), and fiber uniformity (FU), with a significance level of 0.01. Moreover, FL and FS were both negatively correlated with fiber micronaire (FM). In this study, the correlations between FM with and FS were found to be negative but were not significant, which does not agree with previous findings. This deviation could be attributed to the population background used in this study.

It is known that broad sense heritability with high percentage is more useful and very easy to manipulate in MAS. Therefore, the extent of transmission of traits from the parents to the descendants or offspring was determined by level of heritability, hence traits with high broad sense heritability could be easier to manipulate [29]. The broad sense heritability was high for LP (82.65%), FM (91.68%), FR ((86.08%), FY (88.89%), SCI (87.47%), and moderate for BW (68.92%), FL (61.66%), FU (76.42%), FS (76.54%), FE (60.58%), and MI (76.61%). The lowest broad sense heritability was noted for fiber elongation (FE), 60.58%. Similar findings were observed with Jamshed et al. (2016) who found that fiber elongation had the lowest broad sense heritability (27%), whereas other fiber traits were higher, ranging from 80% (FU) to 93% (FL) [11,28].

A total map distance of 4768.098 cM was generated, higher than the most current linkage map with a map distance of the 4450 cM of cotton genome [24]. This is the densest intra-specific map developed in upland cotton. This map could be helpful for further studies in MAS, especially in fine mapping. The average distance of the adjacent markers was 0.92 cM. A_t_ sub-genome spanned 2611.43 cM, and consisted of 3313 markers with 13 LGs. The average marker distance in A_t_ sub-genome was 0.79 cM with a maximum gap of 26.598 cM of the adjacent markers. In D_t_ sub-genome, 13 LGs were assigned which comprised of 1865 markers spanning 2156.67 cM, with an average of 1.156 cM. The maximum gap was 30.082 cM between adjacent loci. Due to the nature of upland cotton genome, mapping QTLs not only for fiber as in this research but for other agronomic traits has been difficult. This is because of the narrow genetic background, which resulted in low diversity of alleles with a significant role in fiber quality traits between two given varieties [30]. Therefore, only few QTLs could be mapped based on two parent crossing populations, which has been verified by previous reports [3,6,31,32,33,34,35,36,37,38,39]. In this current study, a total of 110 QTLs were identified for 11 traits, but only 30 QTLs were consistent in at least two environments. The 30 consistent QTLs were located on 16 chromosomes; A02, A03, A05, A09, A10, A12, D01, D02, D03, D04, D05, D08, D10, D11, D12, and D13 with 2, 1, 2, 3, 2, 1, 1, 1, 4, 1, 2, 2, 2, 1, 4, and 1 QTL respectively. Of the 30 detected QTLs, 11 were located on A_t_ sub-genome while the remaining 19 were located on the D_t_ sub-genome. This finding is consistent with previous reports in which 58 QTLs were found on the A_t_ sub-genome, whereas 107 QTLs were localized on the D_t_ sub-genome [11]. Fifty-eight QTLs were located on the A_t_ sub-genome (Chr01–Chr13), and 73 QTLs on the D_t_ sub-genome [25]. These QTLs explained from 2.03 to 16.85% of phenotypic variation, with an average of 6.26% explained in all five fiber quality traits [40].

Most of the QTLs distributed in the cotton genome revealed the complexity of the cotton genome and arduousness of QTL mapping in cotton. Therefore, comparing our QTLs with other QTLs mapped from previous studies could be of great help in determining the reliability of the QTLs detected [41]. Up to now, 4268 QTLs from 140 publications of cotton have been documented in the collected Cotton QTL Database (available online: http://www2.cottonqtldb.org:8081/index). In this study, the GBS-SNP markers are unique and thus lack common identity with the SSR-based markers. However, five QTL clusters in this investigation were found to have a common bearing to those documented by Said et al. [42], which have been known as one of the strongest references in QTL mapping in recent years. The five QTL clusters were: cluster A07 was identical to c7-cluster-Gh × Gb-4:55–79 cM; cluster A08 had an approximate position of 4.81–110.81 cM, which was similar to c8-cluster-Gh-2:21–31 cM; cluster D01, had an approximate position of 2.21–139.31 cM, similar to c15-cluster-Gh-3:49–68 cM; cluster D02, had an approximate position of 0.01–206.11 cM, similar to c14-cluster-Gh-2:76–91 cM and lastly cluster D08, had an approximate position of 100.71–208.61 cM, nearby to c24-cluster-Gh-2:41–62 cM. The high correlation of the QTLs detected in this study to the previous finding, provides the opportunity for the utilization of these QTLs in MAS to improve the fiber quality of Upland cotton.

Gene Ontology enrichment analysis revealed five genes with very high expression and were linked to three fiber quality traits, FM, FS, and FY. Interestingly, the five genes took their alleles from the parental line known for superior fiber quality CCRI35. This result supported our study. Our findings provide an opportunity in the improvement for fiber quality especially fiber color (FY: fiber yellowness). Cotton fiber development occurs through various stages, namely fiber initiation, elongation, secondary cell wall formation and maturation [43]. Cotton fiber development is controlled by a multi-complex of genes interactions rather than a single gene effect [44,45]. *GhD12G0969* was mainly found to have a functional role in phosphorylation; phosphorylation is a process mediated by protein kinases to activate critical cellular pathways such as metabolism, cell division and cell differentiation during initiation stages in cotton fiber development [46]. In addition, *Gh_D12G0435* was found to be involved in kinase activity; protein kinase activity plays an important role in signal transduction through the phosphorylation process during cotton fiber development [47]. Therefore, the five highly up regulated genes could possibly be the key genes with major functional roles in fiber development and in turn superior quality as evident in the CCRI35, female parent.

## 4. Materials and Methods

### 4.1. Plant Materials, Growth Conditions and Trait Data Collection

The accessions used in this research were, Nan Dan Ba Di Da Hua in Chinese annotation, but for simplicity, we abbreviated the name as (NH), the male parent; it has moderate fiber quality traits but high yielding in fiber [48,49]. The female parent was Zhong35, also with the Chinese name, was then abbreviated as CCRI35; it is known for high fiber quality traits but with moderate yield [9]. The parental lines and 277 F_2:3_ population were evaluated for fiber quality traits and yield components in Anyang research station (36°100′ N, 114°350′ E), Henan province, Yellow River. The field experiment was carried out during summer periods in three consecutive years, 2014, 2015 and 2016. The experimental layout adopted, was complete randomized block design (CRBD) with three replicates. The plot sizes were 5 m long with row spacing of 0.75 m. Fiber quality and yield component traits were collected following the laid down scheme as described by [41]. Fully opened bolls in each sampled plant were collected within the middle region of the plant, 25 bolls were collected from each line for fiber quality and yielded component determination. The balls were ginned for the determination of lint percentage (LP), fiber length (FL), fiber uniformity (FU), fiber micronaire (FM), fiber elongation (FE), fiber strength (FS), fiber reflectance (FR), fiber yellowness [50], spanning consistency index (SCI) and mature index (MI) by the HVI 900 fiber testing system, which was done in our cotton fiber quality testing unit, cotton research institute, Anyang, China. The test conditions were set with temperature at 20 °C and relative humidity of 65%.

### 4.2. Sample Collection, Library Preparation, Sequencing and SNP Genotyping

#### 4.2.1. DNA Extraction, Quantification and Quality Determination

Fresh leaf samples were obtained from each line, together with the two parents and immediately frozen in liquid nitrogen then stored under −80 °C before DNA extraction. DNA of the F_2:3_ populations of 277 individuals and 10 samples for individual parents was extracted by the CTAB method as described by Paterson et al. [51]. Each sample was then crushed separately in liquid nitrogen to fine powder, then immediately added to CTAB solution. In every 100 mg ground tissues, we added 500 µL of CTAB Buffer. The samples were then shaken for 15 min then centrifuged. The centrifuged mixture was then put in a water bath at 60 °C for 30 min. Then, samples were centrifuged for 5 min at 12,000 revolutions per minute (rpm) for 5 min. After centrifuging, the supernatant transferred to a new tube. Then, 5 µL of RNase solution was added to digest RNA and then incubated for 20 min at 32 °C. Equal amount in volume of chloroform/isoamyl alcohol (24:1) was added then shaken for 5 s before centrifuging the samples for 1 min to separate the phases. We pippeted the aqueous upper phase to a new tube; the method was then redone until the upper phase was clear. The upper clear phase was then pipetted into a new tube. DNA samples were later precipitated by adding 70% by volume of ice-cold isopropanol and incubated for 15 min at −20 °C. The condensed DNA samples were then centrifuged at 12,000 rpm for 10 min. The supernatant was then decanted and subsequently washed with 500 µL ice cold 70% ethanol twice then absolute alcohol. DNAs were later dissolved in 20 µL TE buffer (10 mM Tris, pH 8, 1 mM EDTA) [52]. The degradation and contamination of DNA was checked through 1% agarose gels. The purity of DNA was determined by using a Nano Photometer^®^ spectrophotometer (IMPLEN, Westlake Village, CA, USA). The ratio of absorbance at 260 and 280 nm was used to assess the purity of DNA. The DNA samples with the ratio of ~1.8 were then qualified as pure [53]. The concentration of DNA was done by using Qubit^®^ DNA Assay Kit in Qubit^®^ 2.0 Fluorimeter (Life Technologies, Carlsbad, CA, USA). The Qubit^®^ dsDNA HS (High Sensitivity) Assay Kits make DNA quantitation easy and accurate. The kits contain concentrated assay reagent, dilution buffer, and prediluted DNA standards. The reagents were mixed with the buffer solution, and then added 1–20 μL of each DNA samples.

The concentrations were read using the Qubit^®^ Fluorometer (Life Technologies, Carlsbad, CA, USA); only the DNA samples with concentration range of 10 pg/µL to 100 ng/µL were finally used (available online: https://tools.thermofisher.com/content/sfs/manuals/Qubit_dsDNA_HS_Assay_UG.pdf).

#### 4.2.2. GBS Library Preparation, Sequencing and SNP Genotyping

GBS is a low cost and an efficient method of large-scale genotyping, which is based on high-throughput sequencing but with a reduced-representation library (RRL). The following were step by step processes in GBS technique; firstly, we carried out a GBS pre-design experiment to test the accuracy of the GBS protocol and quality of the output data. The enzymes and sizes of restriction fragments were examined by using training data. Three basic criteria were followed: (a) the suitability of the number of tags to the project needs; (b) the homogenous distribution of the enzymatic tags throughout the examined sequences; (c) elimination of redundant tags (repeated tags must be avoided). This was to ensure the effectiveness and accuracy of data obtained from GBS reads; 50 bp was the selection criterion to ensure sequence depth uniformity.

Secondly, we constructed the GBS library using the pre-designed scheme. The genomic DNA of the F_2:3_ population were incubated at 37 °C with MseI Restriction Enzyme obtained from New England BioLabs (Ipswich, MA, USA), NEB, T4 DNA ligase and ATP. MseI Y adapter N containing barcode. Restriction-ligation reactions were activated at 65 °C, followed by digestion for additional restriction enzyme NlaIII at a temperature of 37 °C. The samples were then purified by using Agencourt AMPure XP (Beckman, Brea, CA, USA). Then carried out polymerization chain reaction (PCR) using the purified samples, Phusion Master Mix universal primer and index primer were used to add index, complete i5 and i7 sequence. The Agencourt AMPure XP (Beckman) was used to purify the PCR products, which were pooled then ran through 2% agarose gels. Fragments with 375–400 bp (with indexes and adaptors) in size were obtained by using a Gel Extraction Kit (Qiagen, Hilden, Germany). The isolated fragment products were then purified using Agencourt AMPure XP (Beckman), and finally diluted for sequencing.

GBS analysis was strictly carried out as outlined by Elshire et al. (2011) [54]; integrating 3 of 96-well plates across 288 barcodes for library preparation and sequencing. For SNP calling, the raw sequence data for the 277 F_2:3_ population together with the F_1_generation was processed through the TASSEL 3.0 Genotype By Sequencing (GBS) pipeline [55] using the Gossypium_hirsutum_v1.1.fa as the reference genome [56] which was obtained from Cotton research institute (available online: http://mascotton.njau.edu.cn/info/1054/1118.htm), for alignment and the Burrows–Wheeler Aligner (BWA) mem [57] with default parameters. The output consisted of variant call format (VCF) file version 4.1 [58] including single nucleotide polymorphisms (SNPs) present in at least 40% of the progeny and with a minor allele frequency (MAF) 0.1. Subsequently, the data in variant call format (VCF) was filtered using VCF tools version.1.12a [58] and TASSEL [59] versions 3.0 and 4.0. A total of 93,384 single nucleotide polymorphisms (SNPs) were identified in 277 F_2:3_ population by TASSEL 3.0, then a custom filtering process was applied for alignment. The filtering was based on maintaining sites with a minimum read depth of 6% and 75% completeness by site across progeny and by progeny across sites. Results were obtained as a TASSEL hapmap file.

Finally, using a custom perl script marker heterozygous in the F_1_generations and with a co-dominant segregation ratio of 1:2:1 among the F_2:3_ population were identified using a chi-squared (χ^2^) goodness-of-fit test at α ≤ 0.01. These were reconverted and imported in JoinMap^®^ 4.1 for linkage group generation. A total of 26 LGs were obtained, each linkage group was assigned to its corresponding chromosome by using BLASTN-search (available online: https://blast.ncbi.nlm.nih.gov/Blast.cgi), for the marker sequence.

### 4.3. Data Analysis and Linkage Map Construction

Analysis of variance (ANOVA) was performed by using field phenotype data of the three consecutive seasons 2014, 2015, 2016, and the combine analysis (cb). A mixed procedure was used; the genotypes and the environments were fixed as factors in order to detect the heritability [60]. Post hoc test (Turkey’s) to compare means was done [60]. The broad-sense heritability percentage, H_b_ (%), was calculated for each trait using the formula described by [61].

H = σ^2^G/σ^2^G + (σ^2^e/r)

With σ^2^G is the genotypic variance; σ^2^e: phenotypic variance and r: replication.

Most of the data were analyzed using R software version 3.4.2 (R Foundation for Statistical Computing, Vienna, Austria) [21]. Markers were ordered, rippled, and re-ordered according to pairwise recombination fractions, LOD scores and linkage group length [62]. Linkage group analyses were conducted using Join Map 4.0 [23] with a recombination frequency of 0.40 and a logarithm of odds (LOD) score of 2.5 for the F_2:3_ population. The Kosambi mapping function was employed in the conversion of the recombination frequencies to map distances. Each data point represented the mean of three replications. Fiber quality and yield-related traits such as boll weight (BW), lint percentage (LP), fiber length (FL), fiber uniformity (FU), fiber micronaire (FM), fiber strength (FS), fiber elongation (FE), fiber reflectance (FR), fiber yellowness (FY), spinning consistency index (SCI) and mature index (MI) were used to conduct QTL analysis. The quantitative trait loci (QTLs) were detected using composite interval mapping (CIM) [63] by WinQTL Cartographer version 2.5 [20]. In the CIM mapping method, version 6, forward–reverse regression method with 1 cM walking speed, a probability into and out of the model of *p* = 0.01 and window size set at 10 cM. The LOD [64] threshold value was determined by 1000 permutation tests for all traits and was used to declare the significant QTLs with a significance level of *p* = 0.05. In addition, QTLs with LOD threshold of 2.5 in more than one environment were considered as common QTLs based on the explanation by Lander and Kruglyak [65].

QTL nomenclature was done based on the description by Liang et al. [2]. The proportion of observed phenotypic variance explained by each QTL was estimated by the coefficient of determination *R*^2^ (%) as a percentage. The additive and dominance effects from QTL cartographer results were used to calculate genetic effects (|d/a|). The results were used to classify the QTL as additive effect (Ae) (0–0.20), partial dominant (PD) (0.21–0.80), dominant effect (De) (0.81–1.20) and over dominant (OD) >1.20 according to Stuber et al. (1987) [66]. The graphic presentation of the linkage group and QTLs marked were created by R software version 3.4.2 [21] and Map Chart 2.2 [67], respectively

### 4.4. Gene Mining and Expression Analysis

In this study, only segments of linkage groups associated with significantly detected QTLs were presented. The detected consistent QTLs were used to identify the crucial candidate genes for fiber yield and fiber quality-related traits. The genes identified were searched through the available resources [68] (available online: https://cottonfgd.org). The physical position of the GBS-SNP markers flanking major QTLs for fiber quality and yield-related traits were used to find the gene located in each QTL region. The function of the identified genes was determined through gene annotation. Furthermore, the expression profile of the candidate genes was analyzed by mapping it in the “TM-1”_RNA-seq transcriptome data of cotton (available online: https://cottonfgd.org). The expression values for each gene mined were used to generate the heat map using R-software script [21].

### 4.5. The Gene Ontology Enrichment Analysis BaseD on QTL Clusters

In order to determine the functions of the identified genes, we carried out gene ontology enrichment analysis through online software, Blast2GO (available online: https://www.blast2go.com/). Gene ontology describes the genes in three functional annotations, namely cellular component (CC, biological process (BP) and molecular functions (MF); three functions provide information on the possible roles played by the genes in the plant; of interest were the genes responsible or fiber qualities and yield-related traits. The choice of genes used for GO analysis was based on the genes mined from the two clusters, cluster 1 (D03) and 2 (D12), which had high percentage of phenotypic variation (PV) and heritability (Hb).

## 5. Conclusions

A genetic linkage map comprising of 5178 SNP markers, obtained by the GBS genotyping method, was generated using a 277 F_2:3_ population derived from an intra-specific cross of two tetraploid upland cotton. The map constructed in this study is the highest dense genetic map ever developed from an intra-specific population of the tetraploid upland cotton. The average distance of 0.92 cM was observed between adjacent markers. A total of 110 QTLs were obtained for 11 traits, however, only 30 QTLs were consistent in more than one environment. In addition, we identified 1709 genes that were found in the two main hot spot regions, named as cluster 1 and 2, with four QTLs in each. Out of the 1709 genes, 153 genes exhibited higher expression levels while the rest showed lower expression levels in all stages of fiber development. We further identified five key genes: *Gh_D03G0889*, *Gh_D12G0093*, *Gh_D12G0969*, *Gh_D12G0410*, and *Gh_D12G0435* to be the candidate genes involved in fiber development. This research provides the very first foundation in which future molecular work can be done, such as cloning of the identified genes and/or saturation of the genes to boost the current elite cultivated cotton cultivars.

## Figures and Tables

**Table 1:** ANOVA, broad sense heritability and phenotypic analysis of fiber quality and yield related traits for the two parents and the F_2:3_ population.

Trait	Source	DF	SS	MS	F	Pr > F	H_b_ (%)	P1	P2	P1 − P2	F_2:3_					
											Mean	SD	Max	Min	Skew	Kurt
BW	e	3	327.4	109.1	1.9 × 10^10^	<0.0001	67.5	-	-		4.77	0.73	7.9	1.8	−0.16	0.94
	g	276	728.6	2.6	4.59 × 10^08^	<0.0001										
	g*e	828	710.5	0.9	1.49 × 10^08^	<0.0001										
LP	e	3	1856.7	619	9.5 × 10^10^	<0.0001	82.1	-	-		35.97	3.71	54	16.08	0.09	1.2
	g	276	28,244	102.3	1.57 × 10^10^	<0.0001										
	g*e	828	15,200.7	18.4	2.82 × 10^09^	<0.0001										
FL	e	3	1561.4	521	9.23 × 10^24^	<0.0001	63.6	24.12	21.12	3	26.46	1.2	32.2	21.55	0.94	0.55
	g	276	1530.1	5.5	9.83 × 10^22^	<0.0001										
	g*e	828	1670.7	2	3.58 × 10^22^	<0.0001										
FU	e	3	1200.6	400	9 × 10^23^	<0.0001	77.1	87.23	80.6	6.63	85.14	1.69	89.3	77.8	−0.75	0.12
	g	276	4844.2	17.6	3.95 × 10^22^	<0.0001										
	g*e	828	3321.3	4	9.02 × 10^21^	<0.0001										
FM	e	3	12.700	4	2.19 × 10^25^	<0.0001	92.4	5.61	5.32	0.29	4.57	0.72	6.75	2.2	−0.19	−0.15
	g	276	1406.5	5.1	2.63 × 10^25^	<0.0001										
	g*e	828	320.3	0.4	1.99 × 10^24^	<0.0001										
FS	e	3	2038.7	680	3.37 × 10^25^	<0.0001	76.8	28.97	25.18	3.79	26.19	2.27	36.5	21	0.76	0.75
	g	276	8851.8	32.1	1.59 × 10^24^	<0.0001										
	g*e	828	6155.4	7.4	3.69 × 10^23^	<0.0001										
FE	e	3	10.800	4	2.15 × 10^24^	<0.0001	61.8	6.83	6.87	−0.04	6.51	0.3	8.1	4.5	−1.02	0.02
	g	276	137.1	0.5	2.96 × 10^23^	<0.0001										
	g*e	828	157.5	0.2	1.13 × 10^23^	<0.0001										
FR	e	3	335.500	112	5.58 × 10^22^	<0.0001	84.8	-	-		63.23	2.27	73.6	58.7	0.9	1.22
	g	276	11,577.9	41.9	2.09 × 10^22^	<0.0001										
	g*e	828	5282.3	6.4	3.18 × 10^21^	<0.0001										
FY	e	3	38.2	12.7	8.03 × 10^23^	<0.0001	86.3	-	-		7.83	0.61	9.6	5.5	−0.32	1.19
	g	276	845.6	3.1	1.93 × 10^23^	<0.0001										
	g*e	828	348	0.4	2.65 × 10^22^	<0.0001										
SCI	e	3	24,493.6	8164.5	1.39 × 10^25^	<0.0001	86.6	-	-		122.26	11.57	167	82	0.09	0.97
	g	276	299,967	1086.8	1.85 × 10^24^	<0.0001										
	g*e	828	120,302.4	145.3	2.48 × 10^23^	<0.0001										
MI	e	3	0.5	0.2	6.73 × 10^25^	<0.0001	66.7	-	-		0.81	0.02	0.87	0.75	−0.19	−0.79
	g	276	0.7	0	1.18 × 10^24^	<0.0001										
	g*e	828	0.5	0	2.77 × 10^23^	<0.0001										

P1 = CCRI35: parental female with good fiber quality traits; P2 = NH: good yield fiber; DF: degree of freedom; SS: sum square; MS: mean square; F: F value; H_b_ (%): Broad sense heritability percentage; SD: Standard deviation; Min: Minimum; Max: Maximum; Skew: Skewness; Kurt: Kurtosis, (<0.0001): means significant at level *p* < 0.001.

**Table 2:** Genomic distributions of SNPs markers.

Group	Marker Number	Map Length (cM)	Av Distance (cM)	Max Gap (cM)	<10 cM	>10 cM	Ratio
A01(c1)	448	146.704	0.33	8.505	447	0	1
A02(c2)	705	346.314	0.49	17.848	699	5	0.99
A03(c3)	323	213.937	0.66	17.145	319	3	0.99
A04(c4)	106	203.891	1.92	26.598	99	6	0.93
A05(c5)	378	385.092	1.02	21.198	365	12	0.97
A06(c6)	58	73.063	1.26	15.032	54	3	0.93
A07(c7)	279	205.892	0.74	11.622	276	2	0.99
A08(c8)	69	112.137	1.63	18.894	65	3	0.94
A09(c9)	98	138.501	1.41	19.234	95	2	0.97
A10(c10)	292	202.134	0.69	10.551	287	4	0.98
A11(c11)	51	70.548	1.38	23.241	49	1	0.96
A12(c12)	244	309.608	1.27	19.593	236	7	0.97
A13(c13)	262	203.61	0.78	17.425	256	5	0.98
Subtotal A_t_	3313	2611.43	0.79	26.598	3247	53	0.98
D01(c15)	319	144.092	0.45	6.351	318	0	1
D02(c14)	454	313.268	0.69	14.541	450	3	0.99
D03(c17)	133	170.555	1.28	14.993	131	1	0.98
D04(c22)	114	136.228	1.19	20.275	110	3	0.96
D05(c19)	153	218.788	1.43	27.062	148	4	0.97
D06(c25)	16	79.084	4.94	22.389	12	3	0.75
D07(c16)	169	235.366	1.39	26.041	161	7	0.95
D08(c24)	118	226.688	1.92	20.878	109	8	0.92
D09(c23)	40	136.744	3.42	14.48	33	6	0.83
D10(c20)	80	129.051	1.61	20.539	76	3	0.95
D11(c21)	98	89.782	0.92	27.564	95	2	0.97
D12(c26)	143	194.735	1.36	30.082	135	7	0.94
D13(c18)	28	82.286	2.94	20.917	25	2	0.89
Subtotal D_t_	1865	2156.67	1.156	30.082	1803	49	0.97
TOTAL (A_t_ + D_t_)	5178	4768.1	0.92	30.082	5050	102	0.98

Ratio: number of markers less than (<) 10 cM divided by total number of markers within chromosome. Av: Average; Max: Maximum.

**Table 3:** Consistent QTLs for fiber quality and yield related traits identified in this study.

Trait	QTL	Chr	Start Marker	End Marker	Start Marker (bp)	End Marker (bp)	Start Marker (cM)	End Marker (cM)	Position (cM)	LOD	Ae	De	|d/a|	GA	R^2^ (%)	DPE
FS	qFS-A02_15	A02	mk1761_A02	mk1778_A02	80,488,799	81,766,125	0	17.848	5.01	3.761129	−0.0052	1.5095	290.28846	OD	0.5295	NH
	qFS-A02_cb	A02	mk1761_A02	mk1778_A02	80,488,799	81,766,125	0	17.848	7.01	2.903366	0.0292	0.5368	18.383562	OD	0.0421	CCRI35
	qFS-A02_cb	A02	mk1020_A02	mk1022_A02	827,449	909,242	337.304	346.314	337.11	5.507058	0.2417	0.1944	0.8043029	A	5.6762	CCRI35
SCI	qSCI-A02_15	A02	mk1761_A02	mk1778_A02	80,488,799	8,176,6125	0	17.848	1.01	3.268187	−0.7383	9.0221	12.2201	OD	1.2775	NH
	qSCI-A02_cb	A02	mk1761_A02	mk1778_A02	80,488,799	81,766,125	0	17.848	1.01	2.740499	−0.4142	5.1274	12.379044	OD	0.9695	NH
	qSCI-A02_cb	A02	mk1018_A02	mk1019_A02	822,030	827,340	334.259	337.053	336.31	3.27253	0.9943	4.7528	4.7800463	OD	0.277	CCRI35
FL	qFL-A03_14	A03	mk1922_A03	mk1927_A03	1,863,137	1,863,215	194.163	194.228	194.21	2.54506	0.1746	0.5337	3.056701	OD	0.4959	CCRI35
	qFL-A03_15	A03	mk1989_A03	mk2007_A03	2,881,061	2,936,448	168.853	169.141	168.91	2.599349	0.276	0.1484	0.5376812	PD	3.0929	CCRI35
	qFL-A03_cb	A03	mk11099	mk2084_A03	31,386	666,6259	94.16	102.466	102.21	3.565689	0.148	0.3166	2.1391892	OD	1.445	CCRI35
	qFL-A03_cb	A03	mk2085_A03	mk2087_A03	6,666,473	6,736,164	130.379	130.946	130.41	2.723127	0.1219	0.2515	2.0631665	OD	1.3067	CCRI35
FM	qFM-A05_15	A05	mk2943_A05	mk2952_A05	21,550,988	23,173,778	195.463	206.855	197.51	2.927253	0.1377	−0.2204	1.600581	OD	4.6742	CCRI35
	qFM-A05_cb	A05	mk2943_A05	mk2952_A05	21,550,988	23,173,778	195.463	206.855	197.51	3.040174	0.0684	−0.0932	1.3625731	OD	5.0485	CCRI35
LP	qLP-A05_14	A05	mk2943_A05	mk2952_A05	21,550,988	23,173,778	195.463	206.855	195.51	4.76873	0.9516	−0.8514	0.8947037	D	11.2688	CCRI35
	qLP-A05_cb	A05	mk2943_A05	mk2952_A05	21,550,988	23,173,778	195.463	206.855	195.51	2.673181	0.4032	−1.1877	2.9456845	OD	3.4428	CCRI35
BW	qBW-A09_15	A09	mk6774_A09	mk6775_A09	60,948,395	62,054,979	7.252	15.619	15.61	2.599349	0.1034	0.335	3.2398453	OD	0.2668	CCRI35
	qBW-A09_15	A09	mk6764_A09	mk6772_A09	59,295,756	59,503,467	25.198	26.005	25.21	2.744843	0.0794	0.3894	4.9042821	OD	0.0173	CCRI35
	qBW-A09_cb	A09	mk6764_A09	mk6772_A09	59,295,756	59,503,467	25.198	26.005	25.21	2.831705	0.004	0.2838	70.95	OD	0.6913	CCRI35
FU	qFU-A09_16	A09	mk6410_A09	mk6462_A09	4,242,475	7,339,105	115.638	134.872	117.71	2.62975	−0.0221	0.6762	30.597285	OD	0.1419	NH
	qFU-A09_cb	A09	mk8762	mk6732_A09	13,222	55,126,525	35.486	44.202	40.51	2.586319	−0.0343	0.6945	20.247813	OD	1.3251	NH
SCI	qSCI-A09_15	A09	mk18838	mk6517_A09	64,093	33,530,295	79.726	79.934	79.91	4.60152	2.2915	9.0716	3.9588043	OD	0.5063	CCRI35
	qSCI-A09_15	A09	mk6528_A09	mk6531_A09	37,420,628	37,694,390	87.803	91.571	88.81	3.400651	2.6194	7.2082	2.7518516	OD	1.1267	CCRI35
	qSCI-A09_cb	A09	mk6491_A09	mk6493_A09	15,408,937	17,834,574	73.597	73.647	73.61	2.875136	3.6441	4.9294	1.3527071	OD	0.804	CCRI35
	qSCI-A09_cb	A09	mk18838	mk6517_A09	64,093	33,530,295	79.726	79.934	79.91	3.413681	0.7693	5.3779	6.9906408	OD	0.0048	CCRI35
	qSCI-A09_cb	A09	mk6528_A09	mk6531_A09	37,420,628	37,694,390	87.803	91.571	88.81	2.686211	1.0069	4.6458	4.6139637	OD	0.1298	CCRI35
FM	qFM-A10_15	A10	mk7018_A10	mk7020_A10	15,617,318	15,617,342	165.83	165.941	165.91	2.421281	0.0767	−0.322	4.1981747	OD	2.1861	CCRI35
	qFM-A10_15	A10	mk11965	mk6991_A10	8852	12,815,805	171.292	171.66	171.31	3.339848	0.0919	−0.3583	3.898803	OD	3.1176	CCRI35
	qFM-A10_cb	A10	mk18875	mk18876	355	389	58.679	59.519	58.71	2.912052	0.0306	−0.1769	5.7810458	OD	2.1098	CCRI35
	qFM-A10_cb	A10	mk11965	mk6991_A10	8852	12,815,805	171.292	171.66	171.31	2.779587	0.0402	−0.1538	3.8258706	OD	2.621	CCRI35
FS	qFS-A10_16	A10	mk19550	mk7479_A10	62,129	69,679,150	93.736	94.208	93.81	2.877307	−0.4206	0.0644	0.1531146	A	7.1603	NH
	qFS-A10_16	A10	MulMa189-m_A10	mk7438_A10	65,789,277	67,378,405	106.543	109.182	106.81	3.170467	0.0048	0.3517	73.270833	OD	0.4704	CCRI35
	qFS-A10_cb	A10	mk6982_A10	mk6986_A10	1,111,5569	12,645,781	177.095	185.191	177.11	3.676439	−0.1987	0.1727	0.8691495	D	6.206	NH
FE	qFE-A12_14	A12	mk9173_A12	mk9187_A12	79,355,806	81,262,301	23.53	38.305	31.51	2.54506	−0.0177	0.1178	6.6553672	OD	3.0701	NH
	qFE-A12_16	A12	mk8958_A12	mk8961_A12	59,776,633	6,102,5182	161.739	173.999	163.81	2.838219	0.0006	0.0458	76.333333	OD	0.0468	CCRI35
FE	qFE-D01_15	D01	mk10708_D01	mk10809_D01	42,734,090	44,178,280	105.061	106.117	106.11	2.551574	−0.0001	0.1905	1905	OD	0.4005	NH
	qFE-D01_cb	D01	mk10832_D01	MulMa266-m_D01	46,916,080	51,199,148	77.706	82.287	79.71	4.141151	0.0444	0.0955	2.1509009	OD	0.7434	CCRI35
	qFE-D01_cb	D01	mk10708_D01	mk10709_D01	42,734,090	42,734,155	106.1	106.117	106.11	2.998914	0.0009	0.0961	106.77778	OD	0.4425	CCRI35
SCI	qSCI-D02_15	D02	mk11587_D02	mk11605_D02	51,060,053	5,122,5737	120.178	120.621	120.61	2.690554	1.4485	−6.7494	4.6595789	OD	2.84	CCRI35
	qSCI-D02_cb	D02	mk11595_D02	mk11603_D02	51,118,850	51,193,929	115.939	116.818	116.01	2.521173	0.2862	−4.485	15.67086	OD	1.8379	CCRI35
BW	qBW-D03_15	D03	mk12041_D03	mk12042_D03	2,290,601	2,894,288	22.573	37.566	30.61	2.644951	−0.0899	0.4957	5.5139043	OD	2.9343	NH
	qBW-D03_16	D03	mk12031_D03	mk12032_D03	1,002,704	1,037,917	3.775	9.172	3.81	2.870793	0.0006	0.4782	797	OD	0.7748	CCRI35
FM	qFM-D03_15	D03	mk12142_D03	mk12159_D03	36,697,656	3,861,6587	125.137	134.584	130.21	7.52443	0.2667	0.1477	0.5538058	PD	8.7707	CCRI35
	qFM-D03_15	D03	mk12152_D03	mk12159_D03	37,665,167	38,616,587	134.584	141.665	136.01	7.685125	0.2661	0.0664	0.2495303	PD	10.0325	CCRI35
	qFM-D03_15	D03	mk12153_D03	mk12158_D03	37,668,262	37,938,158	143.823	145.337	144.81	6.304017	0.2329	0.0922	0.3958781	PD	7.4939	CCRI35
	qFM-D03_cb	D03	mk12085_D03	mk12086_D03	25,573,334	25,700,132	87.285	87.516	87.31	4.95114	0.0975	0.0212	0.2174359	PD	6.3845	CCRI35
	qFM-D03_cb	D03	mk12119_D03	mk12123_D03	30,535,745	30,566,883	95.393	95.724	95.41	6.644951	0.1133	0.0204	0.180053	A	8.5806	CCRI35
	qFM-D03_cb	D03	mk12108_D03	mk12115_D03	2,763,8133	29,511,299	101.991	103.218	101.31	4.827362	0.095	0.061	0.6421053	PD	5.0024	CCRI35
FY	qFY-D03_15	D03	mk12142_D03	mk12159_D03	36,697,656	38,616,587	125.137	134.584	130.21	2.610206	0.1828	−0.0298	0.1630197	A	4.7288	CCRI35
	qFY-D03_15	D03	mk12154_D03	mk12155_D03	37,676,414	37,682,981	141.665	142.221	141.71	3.806732	0.184	−0.1844	1.0021739	D	6.9294	CCRI35
	qFY-D03_15	D03	mk12158_D03	mk12161_D03	37,938,158	39,407,242	145.337	150.198	148.31	4.210641	0.2038	−0.2276	1.1167812	D	8.0817	CCRI35
	qFY-D03_cb	D03	mk12109_D03	mk12111_D03	27,707,667	29,136,194	105.804	106.491	105.81	2.571118	0.0879	−0.0931	1.0591581	D	4.7115	CCRI35
LP	qLP-D03_15	D03	mk12142_D03	mk12159_D03	36,697,656	38,616,587	125.137	134.584	129.21	9.233442	1.7674	−0.2922	0.1653276	A	15.4584	CCRI35
	qLP-D03_15	D03	mk12152_D03	mk12160_D03	37,665,167	38,832,736	134.986	141.665	138.01	8.214984	1.6425	−0.399	0.2429224	PD	13.9321	CCRI35
	qLP-D03_16	D03	mk12152_D03	mk12160_D03	37,665,167	38,832,736	134.986	141.665	138.01	5.439739	1.4767	−0.9737	0.6593756	PD	9.8343	CCRI35
	qLP-D03_16	D03	mk12153_D03	mk12158_D03	37,668,262	37,938,158	143.823	145.337	144.81	4.621064	1.2806	−1.0766	0.8406997	D	7.9495	CCRI35
	qLP-D03_cb	D03	mk12152_D03	mk12160_D03	37,665,167	38,832,736	134.986	141.665	139.01	8.169381	1.2356	−0.1607	0.1300583	A	13.3867	CCRI35
	qLP-D03_cb	D03	mk12158_D03	mk12161_D03	37,938,158	39,407,242	145.337	150.198	147.31	7.090119	1.1317	−0.3152	0.278519	PD	12.0557	CCRI35
FR	qFR-D04_15	D04	MulMa448_D04	MulMa451_D04	49,867,798	50,187,192	4.736	8.337	5.01	2.660152	−0.1841	−1.5832	8.5996741	OD	0.0079	NH
	qFR-D04_cb	D04	MulMa448_D04	MulMa451_D04	49,867,798	50,187,192	4.736	8.337	5.01	2.781759	−0.227	−0.8557	3.7696035	OD	0.6158	NH
FM	qFM-D05_15	D05	mk12822_D05	mk12824_D05	30,214,244	30,216,527	152.95	153.434	153.01	5.061889	0.2063	0.008	0.0387785	A	6.8075	CCRI35
	qFM-D05_cb	D05	MulMa463-m_D05	mk12861_D05	30,373,354	31,354,896	144.668	148.809	146.71	4.021716	0.08	−0.1214	1.5175	OD	6.6096	CCRI35
	qFM-D05_cb	D05	mk12822_D05	mk12824_D05	30,214,244	30,216,527	152.95	153.434	153.41	4.627579	0.0886	−0.0879	0.9920993	D	7.3098	CCRI35
SCI	qSCI-D05_15	D05	MulMa463-m_D05	mk12861_D05	30,373,354	31,354,896	144.668	148.809	144.71	2.566775	−2.7495	3.5799	1.3020185	OD	4.5444	NH
	qSCI-D05_cb	D05	MulMa463-m_D05	mk12861_D05	30,373,354	31,354,896	144.668	148.809	144.71	2.579805	−1.778	1.3725	0.7719348	PD	4.7561	NH
FR	qFR-D08_15	D08	mk15992_D08	mk15995_D08	56,628,640	56,628,844	181.945	182.082	182.01	2.655809	0.587	−0.2932	0.4994889	PD	4.6002	CCRI35
	qFR-D08_cb	D08	mk15992_D08	mk15995_D08	56,628,640	56,628,844	181.945	182.082	182.01	3.441911	0.408	−0.2977	0.7296569	PD	6.2705	CCRI35
FL	qFL-D08_14	D08	MulMa514_D08	mk16004_D08	54,937,781	58,533,805	187.587	196.342	196.31	3.583062	0.3322	−0.0152	0.0457556	A	5.8804	CCRI35
	qFL-D08_cb	D08	MulMa514_D08	mk16004_D08	54,937,781	5,853,3805	187.587	196.342	196.31	3.639522	0.1799	0.0892	0.495831	PD	4.9405	CCRI35
	qFL-D08_cb	D08	mk16017_D08	mk16020_D08	59,691,087	59,698,388	208.553	208.76	208.61	3.255157	0.1595	0.1876	1.1761755	D	3.2928	CCRI35
FE	qFE-D10_14	D10	mk17141_D10	MulMa593-m_D10	56,817,432	56,887,380	102.161	106.55	106.21	2.523344	−0.0328	0.082	2.5	OD	3.7527	NH
	qFE-D10_15	D10	MulMa575-m_D10	MulMa581_D10	24,793,863	24,918,141	1.604	2.047	1.91	2.959826	0.0751	0.0306	0.4074567	PD	3.9966	CCRI35
FL	qFL-D10_14	D10	mk17141_D10	MulMa593-m_D10	56,817,432	56,887,380	102.161	106.55	105.21	3.411509	−0.1394	0.7384	5.2969871	OD	3.6287	NH
	qFL-D10_cb	D10	mk2492	MulMa366-m	519	33,037	78.155	98.694	83.21	2.614549	−0.045	0.464	10.311111	OD	2.0017	NH
FL	qFL-D11_16	D11	mk17462_D11	mk17463_D11	15,695,804	15,711,598	88.706	88.935	88.71	2.831705	0.0011	0.1765	160.45455	OD	0.8561	CCRI35
	qFL-D11_cb	D11	mk17464_D11	mk17514_D11	15,711,711	21,298,890	61.142	88.706	82.21	3.079262	−0.0461	0.5185	11.247289	OD	2.2603	NH
FS	qFS-D12_14	D12	mk18221_D12	MulMa605_D12	50,554,371	5,129,3378	148.99	160.984	153.01	3.270358	0.0077	0.8261	107.28571	OD	0.6079	CCRI35
	qFS-D12_15	D12	mk17994_D12	mk17997_D12	3,798,8313	3,8143,957	65.608	66.056	65.61	3.743757	0.3487	−0.7277	2.0868942	OD	5.7325	CCRI35
	qFS-D12_cb	D12	mk19853	mk17913_D12	101,319	13,479,261	16.392	16.946	16.41	4.049946	0.1807	−0.3037	1.6806862	OD	6.3446	CCRI35
FY	qFY-D12_15	D12	mk17995_D12	mk18057_D12	38,058,755	41,722,495	66.861	96.943	70.91	2.677524	0.047	−0.4721	10.044681	OD	2.0175	CCRI35
	qFY-D12_cb	D12	mk1009	mk17992_D12	18,989	37,732,030	66.348	66.861	66.41	3.252986	0.0211	−0.2739	12.981043	OD	1.9523	CCRI35
SCI	qSCI-D12_14	D12	mk18202_D12	mk18207_D12	48,411,387	48,718,111	132.168	133.627	133.61	3.14658	1.3176	−0.8128	0.6168792	PD	6.2241	CCRI35
	qSCI-D12_15	D12	mk19857	mk17916_D12	117,142	15,801,265	23.692	24.245	23.71	3.072747	2.636	4.6094	1.7486343	OD	1.4248	CCRI35
FL	qFL-D12_14	D12	mk18210_D12	mk18214_D12	48,923,084	49133419	135.788	138.113	135.81	2.851249	0.281	−0.216	0.7686833	PD	5.2886	CCRI35
	qFL-D12_cb	D12	mk18221_D12	MulMa605_D12	50,554,371	51,293,378	148.99	160.984	158.01	2.896851	0.1755	0.1739	0.9908832	D	3.2967	CCRI35
	qFL-D12_cb	D12	MulMa604-m_D12	mk18232_D12	51,286,859	52,905,207	161.79	172.395	165.81	2.773073	0.1653	0.1375	0.8318209	D	3.0451	CCRI35
FL	qFL-D13_14	D13	mk18377_D13	MulMa619_D13	4,171,037	34,392,983	36.052	56.969	46.11	3.237785	0.1336	−0.7846	5.8727545	OD	3.0376	CCRI35
	qFL-D13_15	D13	mk18378_D13	mk18379_D13	4,310,490	4,329,364	64.756	65.409	64.81	2.529859	0.2624	0.2724	1.0381098	D	2.1912	CCRI35
	qFL-D13_cb	D13	mk18516_D13	mk18533_D13	41,759,681	47,859,575	2.023	10.429	9.01	3.051031	0.17	−0.0572	0.3364706	PD	5.3187	CCRI35
	qFL-D13_cb	D13	mk20382	mk18378_D13	141	4,310,490	65.409	72.523	65.41	3.072747	0.1705	−0.0658	0.3859238	PD	5.4653	CCRI35

LOD: logarithm of odds; 0 < Ae (additive effect) < 0.20; 0.21 < PD (partial dominance) < 0.80; 0.81 < De (dominance effect) < 1.20; OD (over dominance) > 1.20; |d/a| = De/Ae; GA: gene action; DPE: direction of phenotypic explanation. For traits meaning see Figure 1 or Figure 2.

## Supplementary Materials

Supplementary materials can be found at http://www.mdpi.com/1422-0067/19/2/441/s1.
