# Supplementary material for: QTL Mapping of Fiber Quality and Yield-Related Traits in an Intra-Specific Upland Cotton Using Genotype by Sequencing (GBS)
Source: Int J Mol Sci. 2018 Feb 1;19(2):441. doi: 10.3390/ijms19020441 (PMC5855663; doi:10.3390/ijms19020441)
Supplement: Supplementary file 1 [file ijms-19-00441-s001.zip › Supplementary files/Figure S1 Total QTLs (110) found in this study, asterisk means consistent QTLs.pdf]

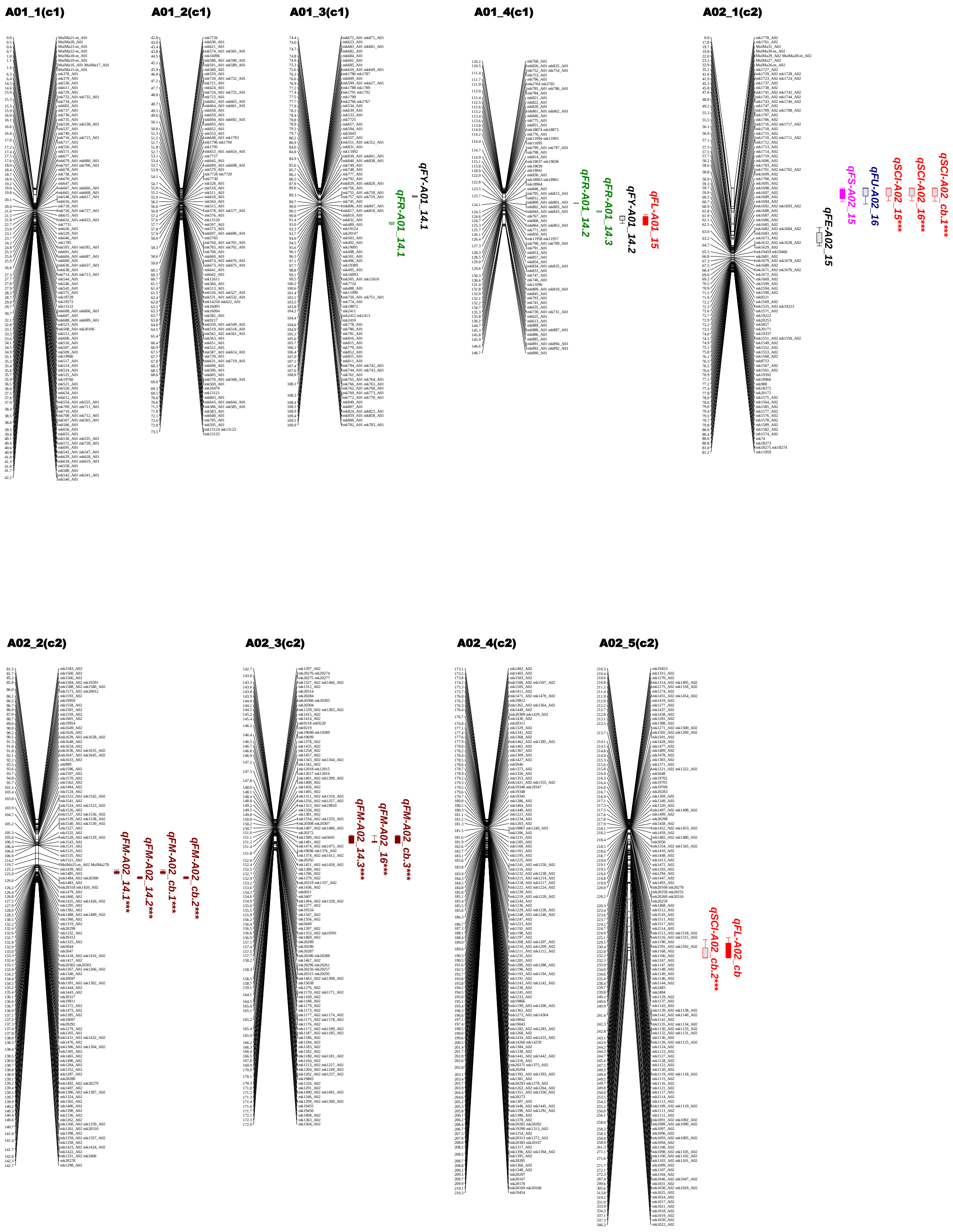

A01_1(c1)
A01_2(c1)
A01_3(c1)
A01_4(c1)
A02_1(c2)
qFY-A01_14.1
qFR-A01_14.1
qFR-A01_14.2
qFR-A01_14.3
qFY-A01_14.2
qFL-A01_15
qFE-A02_15
qFS-A02_15
qFU-A02_16
qSCI-A02_15***
qSCI-A02_16***
qSCI-A02_cb.1***
A02_2(c2)
A02_3(c2)
A02_4(c2)
A02_5(c2)
qFM-A02_14.1***
qFM-A02_14.2***
qFM-A02_cb.1***
qFM-A02_cb.2***
qFM-A02_14.3***
qFM-A02_16***
qFM-A02_cb.3***
qSCI-A02_cb.2***
qFL-A02_cb

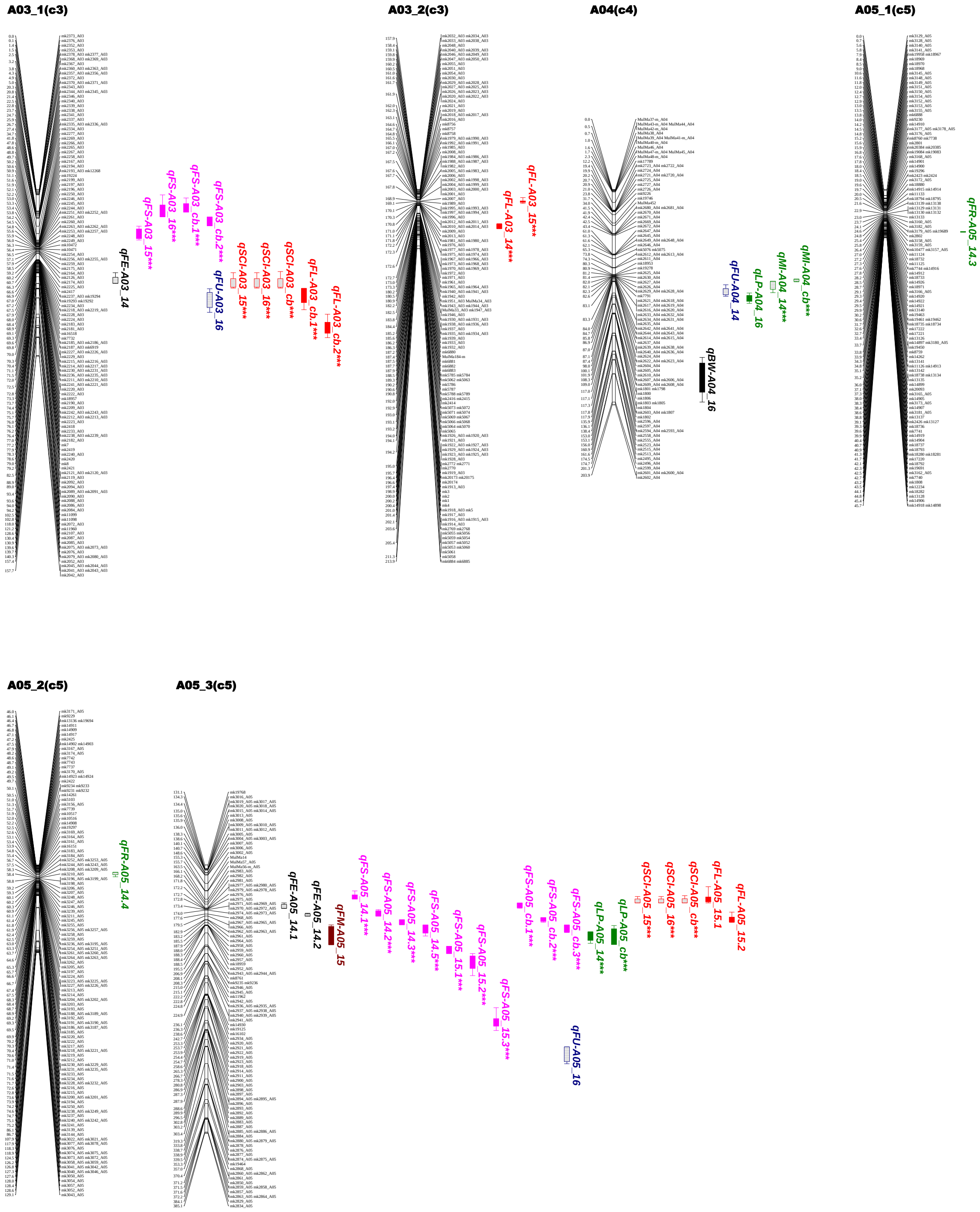
A03_1(c3)
A03_2(c3)
A04(c4)
A05_1(c5)
A05_2(c5)
A05_3(c5)
qFE-A03_14
qFS-A03_15***
qFS-A03_16***
qFS-A03_cb.1***
qFS-A03_cb.2***
qFU-A03_16
qSCI-A03_15***
qSCI-A03_16***
qSCI-A03_cb***
qFL-A03_cb.1***
qFL-A03_cb.2***
qFL-A03_14***
qFL-A03_15***
qFU-A04_14
qLP-A04_16
qMI-A04_14***
qMI-A04_cb***
qBW-A04_16
qFR-A05_14.3
qFR-A05_14.4
qFE-A05_14.1
qFE-A05_14.2
qFM-A05_15
qFS-A05_14.1***
qFS-A05_14.2***
qFS-A05_14.3***
qFS-A05_14.5***
qFS-A05_15.1***
qFS-A05_15.2***
qFS-A05_15.3***
qFS-A05_cb.1***
qFS-A05_cb.2***
qFS-A05_cb.3***
qLP-A05_14***
qLP-A05_cb***
qFU-A05_16
qSCI-A05_15***
qSCI-A05_16***
qSCI-A05_cb***
qFL-A05_15.1
qFL-A05_15.2

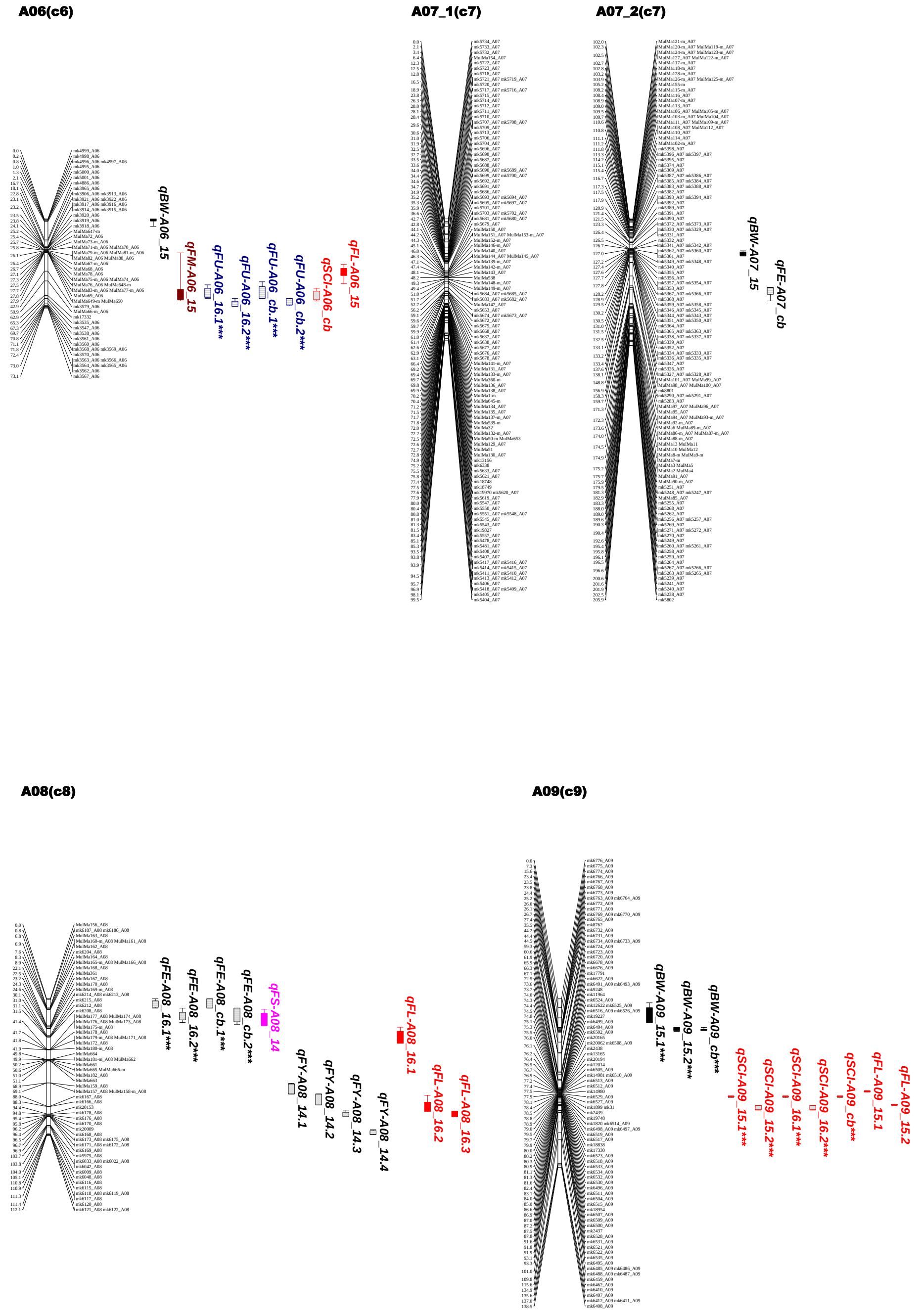
A06(c6)
qBW-A06_15
qFM-A06_15
qFU-A06_16.1***
qFU-A06_16.2***
qFU-A06_cb.1***
qFU-A06_cb.2***
qSCI-A06_cb
qFL-A06_15
A07_1(c7)
A07_2(c7)
qBW-A07_15
qFE-A07_cb
A08(c8)
qFE-A08_16.1***
qFE-A08_16.2***
qFE-A08_cb.1***
qFE-A08_cb.2***
qFS-A08_14
qFY-A08_14.1
qFY-A08_14.2
qFY-A08_14.3
qFY-A08_14.4
qFL-A08_16.1
qFL-A08_16.2
qFL-A08_16.3
A09(c9)
qBW-A09_15.1***
qBW-A09_15.2***
qBW-A09_cb***
qSCI-A09_15.1***
qSCI-A09_15.2***
qSCI-A09_16.1***
qSCI-A09_16.2***
qSCI-A09_cb***
qFL-A09_15.1
qFL-A09_15.2

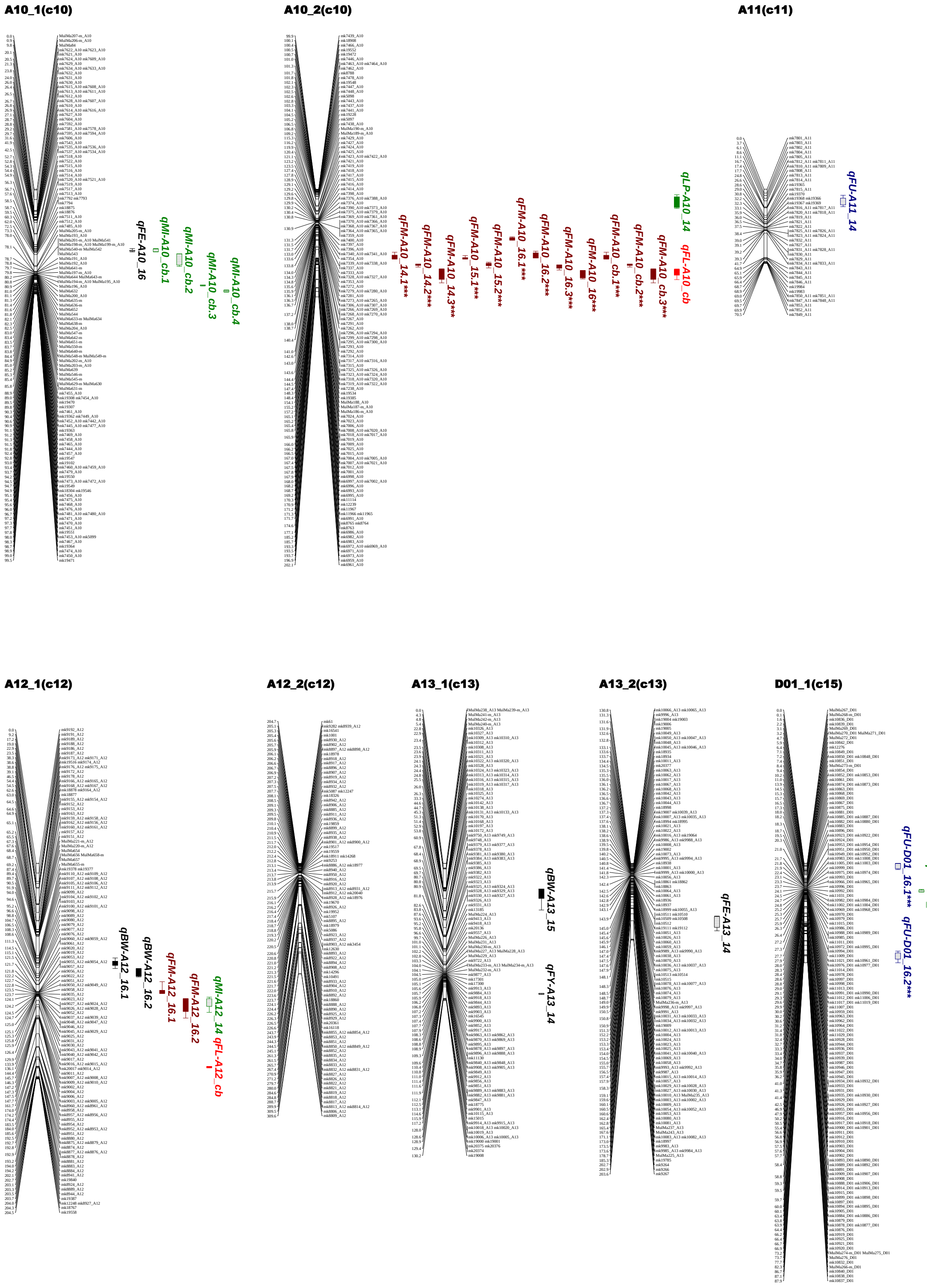
A10_1(c10)
A10_2(c10)
A11(c11)
qFE-A10_16
qMI-A10_cb.1
qMI-A10_cb.2
qMI-A10_cb.3
qMI-A10_cb.4
qFM-A10_14.1***
qFM-A10_14.2***
qFM-A10_14.3***
qFM-A10_15.1***
qFM-A10_15.2***
qFM-A10_16.1***
qFM-A10_16.2***
qFM-A10_16.3***
qFM-A10_16***
qFM-A10_cb.1***
qFM-A10_cb.2***
qFM-A10_cb.3***
qFL-A10_cb
qLP-A10_14
qFU-A11_14
A12_1(c12)
A12_2(c12)
A13_1(c13)
A13_2(c13)
D01_1(c15)
qBW-A12_16.1
qBW-A12_16.2
qFM-A12_16.1
qFM-A12_16.2
qMI-A12_14
qFL-A12_cb
qBW-A13_15
qFY-A13_14
qFE-A13_14
qFU-D01_16.1***
qFU-D01_16.2***
qMI-D01_14

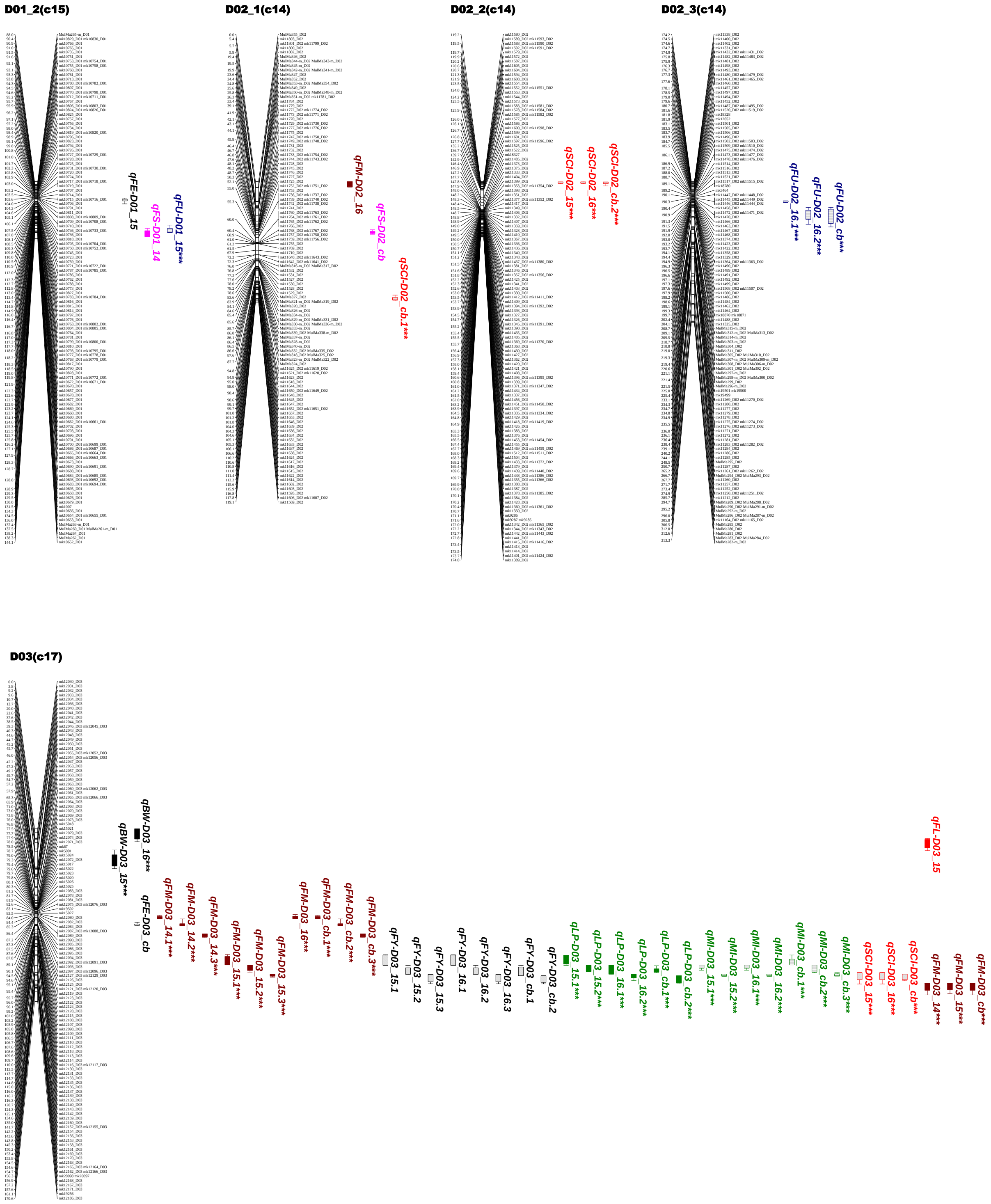

D01_2(c15)
D02_1(c14)
D02_2(c14)
D02_3(c14)
qFE-D01_15
qFS-D01_14
qFU-D01_15***
qFM-D02_16
qFS-D02_cb
qSCI-D02_cb.1***
qSCI-D02_15***
qSCI-D02_16***
qSCI-D02_cb.2***
qFU-D02_16.1***
qFU-D02_16.2***
qFU-D02_cb***
D03(c17)
qBW-D03_15***
qBW-D03_16***
qFE-D03_cb
qFM-D03_14.1***
qFM-D03_14.2***
qFM-D03_14.3***
qFM-D03_15.1***
qFM-D03_15.2***
qFM-D03_15.3***
qFM-D03_16***
qFM-D03_cb.1***
qFM-D03_cb.2***
qFM-D03_cb.3***
qFY-D03_15.1
qFY-D03_15.2
qFY-D03_15.3
qFY-D03_16.1
qFY-D03_16.2
qFY-D03_16.3
qFY-D03_cb.1
qFY-D03_cb.2
qLP-D03_15.1***
qLP-D03_15.2***
qLP-D03_16.1***
qLP-D03_16.2***
qLP-D03_cb.1***
qLP-D03_cb.2***
qMI-D03_15.1***
qMI-D03_15.2***
qMI-D03_16.1***
qMI-D03_16.2***
qMI-D03_cb.1***
qMI-D03_cb.2***
qMI-D03_cb.3***
qSCI-D03_15***
qSCI-D03_16***
qSCI-D03_cb***
qFM-D03_14***
qFM-D03_15***
qFM-D03_cb***
qFL-D03_15

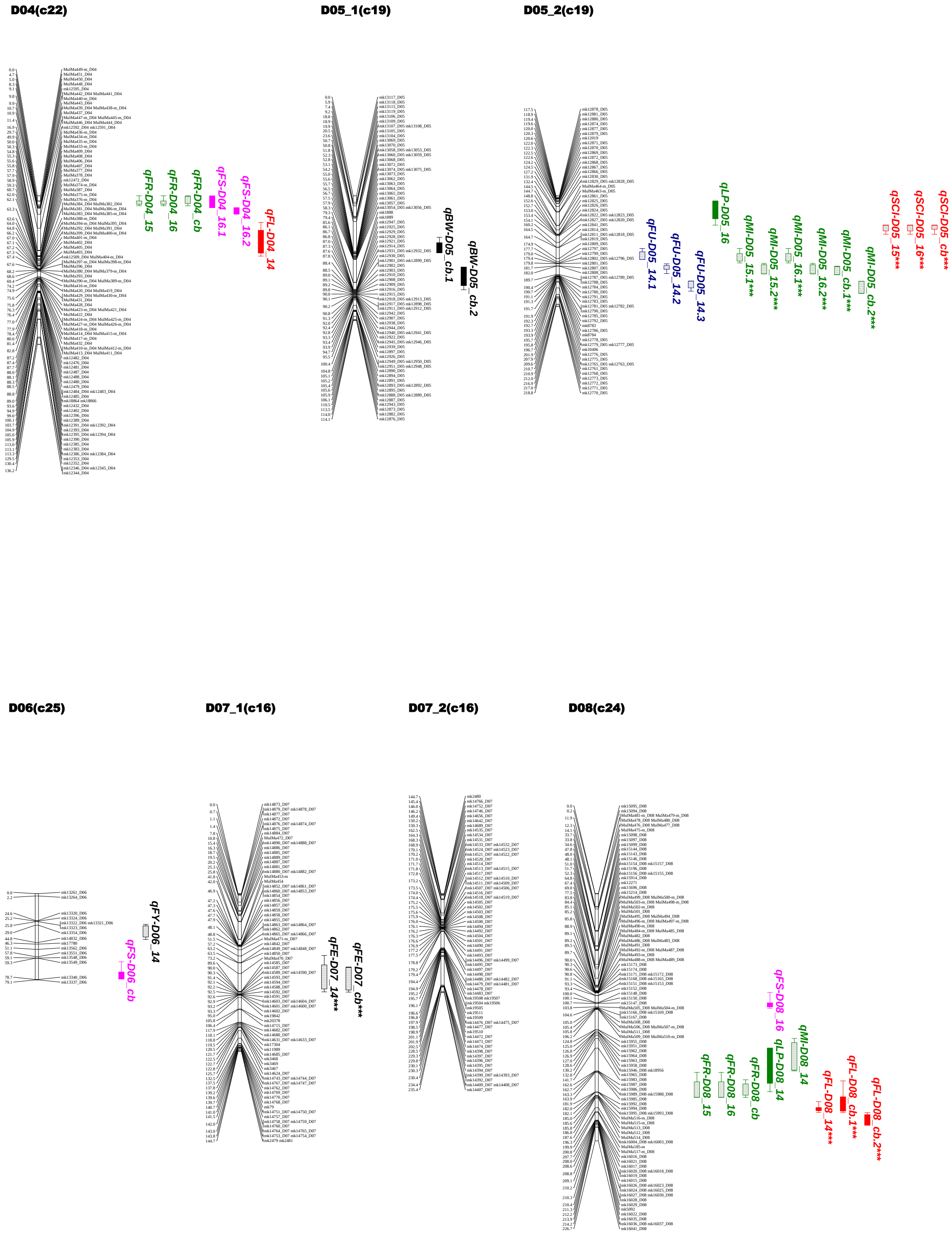
D04(c22)
qFR-D04_15
qFR-D04_16
qFR-D04_cb
qFS-D04_16.1
qFS-D04_16.2
qFL-D04_14
D05_1(c19)
qBW-D05_cb.1
qBW-D05_cb.2
D05_2(c19)
qFU-D05_14.1
qFU-D05_14.2
qFU-D05_14.3
qLP-D05_16
qMI-D05_15.1***
qMI-D05_15.2***
qMI-D05_16.1***
qMI-D05_16.2***
qMI-D05_cb.1***
qMI-D05_cb.2***
qSCI-D05_15***
qSCI-D05_16***
qSCI-D05_cb***
D06(c25)
qFY-D06_14
qFS-D06_cb
D07_1(c16)
qFE-D07_14***
qFE-D07_cb***
D07_2(c16)
D08(c24)
qFS-D08_16
qMI-D08_14
qLP-D08_14
qFR-D08_15
qFR-D08_16
qFR-D08_cb
qFL-D08_14***
qFL-D08_cb.1***
qFL-D08_cb.2***

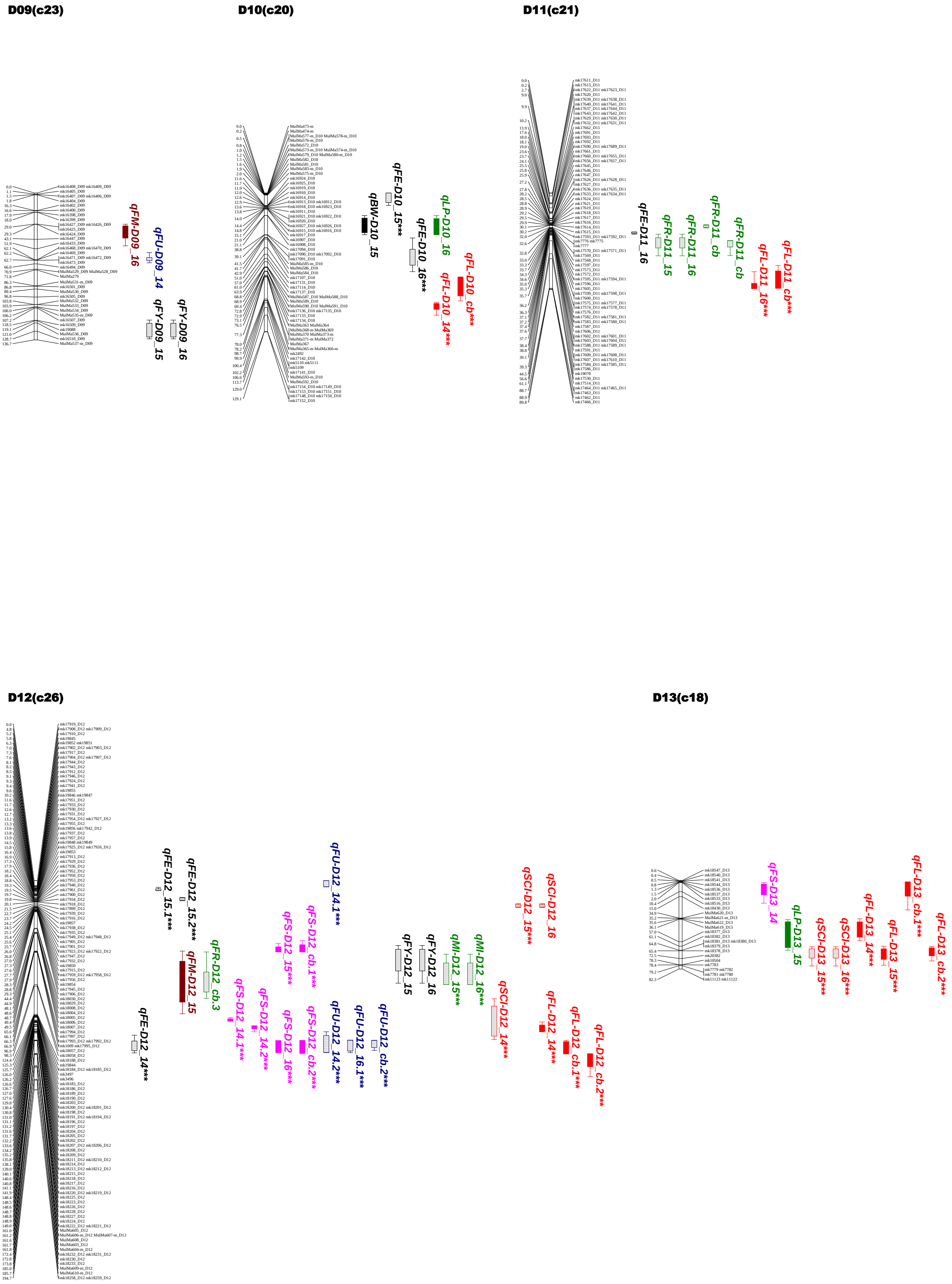

Figure S1: Total QTLs (110) found in this study, asterisk means consistent QTLs
